# Royleanone Derivatives From *Plectranthus* spp. as a Novel Class of P-Glycoprotein Inhibitors

**DOI:** 10.3389/fphar.2020.557789

**Published:** 2020-11-17

**Authors:** Catarina Garcia, Vera M. S. Isca, Filipe Pereira, Carlos M. Monteiro, Epole Ntungwe, Francisco Sousa, Jelena Dinic, Suvi Holmstedt, Amílcar Roberto, Ana Díaz-Lanza, Catarina P. Reis, Milica Pesic, Nuno R. Candeias, Ricardo J. Ferreira, Noélia Duarte, Carlos A. M. Afonso, Patrícia Rijo

**Affiliations:** ^1^ Center for Research in Biosciences & Health Technologies (CBIOS), Universidade Lusófona de Humanidades e Tecnologias, Lisboa, Portugal; ^2^ Department of Biomedical Sciences, Faculty of Pharmacy, University of Alcalá, Alcalá de Henares, Spain; ^3^ Instituto de Investigação do Medicamento (iMed.ULisboa), Faculty of Pharmacy, Universidade de Lisboa, Lisboa, Portugal; ^4^ Institute for Biological Research “Siniša Stanković“- National Institute of Republic of Serbia, University of Belgrade, Belgrade, Serbia; ^5^ Faculty of Engineering and Natural Sciences, Tampere University, Tampere, Finland; ^6^ LAQV-REQUIMTE, Department of Chemistry, University of Aveiro, Aveiro, Portugal; ^7^ Science for Life Laboratory, Department of Cell and Molecular Biology, Uppsala University, Uppsala, Sweden

**Keywords:** *Plectranthus*, Diterpenes, Royleanones, stability, *Artemia salina*, P-pg activity

## Abstract

Cancer is among the leading causes of death worldwide. One of the most challenging obstacles in cancer treatment is multidrug resistance (MDR). Overexpression of P-glycoprotein (P-gp) is associated with MDR. The growing incidence of cancer and the development of MDR drive the search for novel and more effective anticancer drugs to overcome the MDR problem. Royleanones are natural bioactive compounds frequently found in *Plectranthus* spp. The cytotoxic diterpene 6,7-dehydroroyleanone (**1**) is the main component of the *P. madagascariensis* (Pers.) Benth. essential oil, while 7α-acetoxy-6β-hydroxyroyleanone (**2**) can be isolated from acetonic extracts of *P. grandidentatus* Gürke. The reactivity of the natural royleanones **1** and **2** was explored to obtain a small library of new P-gp inhibitors. Four new derivatives (6,7-dehydro-12-*O*-*tert*-butyl-carbonate-royleanone (**20**), 6,7-dehydro-12-*O*-methylroyleanone (**21**), 6,7-dehydro-12-*O*-benzoylroyleanone (**22**), and 7α-acetoxy-6β-hydroxy-12-*O*-benzoylroyleanone (**23**) were obtained as pure with overall modest to excellent yields (21–97%). P-gp inhibition potential of the derivatives **20**–**23** was evaluated in human non-small cell lung carcinoma NCI-H460 and its MDR counterpart NCI-H460/R with the P-gp overexpression, through MTT assay. Previously prepared diterpene 7α-acetoxy-6β-benzoyloxy-12-*O*-(4-chloro)benzoylroyleanone (**4**), has also been tested. The P-gp inhibiting effects of compounds **1**–**4** were also assessed through a Rhodamine 123 accumulation assay. Derivatives **4** and **23** have significant P-gp inhibitory potential. Regarding stability and P-gp inhibition potential, results suggest that the formation of benzoyl esters is a more convenient approach for future derivatives with enhanced effect on the cell viability decrease. Compound **4** presented higher anti-P-gp potential than the natural diterpenes **1**, **2,** and **3**, with comparable inhibitory potential to Dexverapamil. Moreover, derivative **4** showed the ability to sensitize the resistant NCI-H460/R cells to doxorubicin.

## Introduction

Cancer is among the leading causes of death worldwide with an estimated 18.1 million new cancer cases and 9.6 million cancer deaths in 2018 (Bray et al., 2018). One of the most challenging obstacles in cancer treatment is multidrug resistance (MDR). MDR is responsible for over 90% of deaths in cancer patients receiving traditional chemotherapeutics or novel targeted drugs. MDR can be caused by numerous mechanisms in cancer cells, such as activation of DNA repair mechanisms, elevated metabolism of xenobiotics, genetic factors, and increased activity of drug efflux pumps. (Bukowski et al., 2020). Nonetheless, the most common mechanism of MDR is the overexpression of drug efflux transporters of the ATP binding cassette (ABC) family. Three major proteins of the ABC family, namely P-glycoprotein (P-gp, also referred to as MDR1), MDR-associated protein 1 (MRP1), and breast cancer resistance protein (BCRP), were shown to play a critical role in MDR (Mohammad et al., 2018). These efflux pumps are present in the cell membrane of a variety of normal tissues and have a protecting role against xenobiotic substances and toxic compounds. Therefore, they can interfere with drug administration, by reducing the intracellular accumulation of many anticancer drugs to sub-therapeutic levels, thus decreasing or abolishing chemotherapy efficacy (Nanayakkara et al., 2018). P-gp is the best-studied drug efflux pump of the family of ABC transporters. Cancer cells upregulate P-gp expression as an adaptive response to evade chemotherapy mediated cell death. This process leads to resistance against the currently available anti-cancer drugs in many different types of cancers (Sharom, 2007; Nanayakkara et al., 2018; Robinson and Tiriveedhi, 2020) Consequently, the development of P-gp inhibitors is gaining much importance in numerous research works. Several P-gp inhibitors have been discovered by *in silico* and pre-clinical studies. Although P-gp inhibitors showed high efficacy *in vitro* and *in vivo* studies, very few have successfully passed all phases of the clinical trials and none of them have been approved by the U.S. Food and Drug Administration (FDA) for clinical use in cancer treatment (Nanayakkara et al., 2018; Robinson and Tiriveedhi, 2020). After three generations of P-gp inhibitors, a fourth generation comprised of nature-originated compounds has emerged (Dinić et al., 2020). Therefore, identification of natural compounds that can exert anticancer effects and at the same time revert the MDR contributes to the efforts of the cancer research community to combat this multifactorial disease.

**FIGURE 1:**
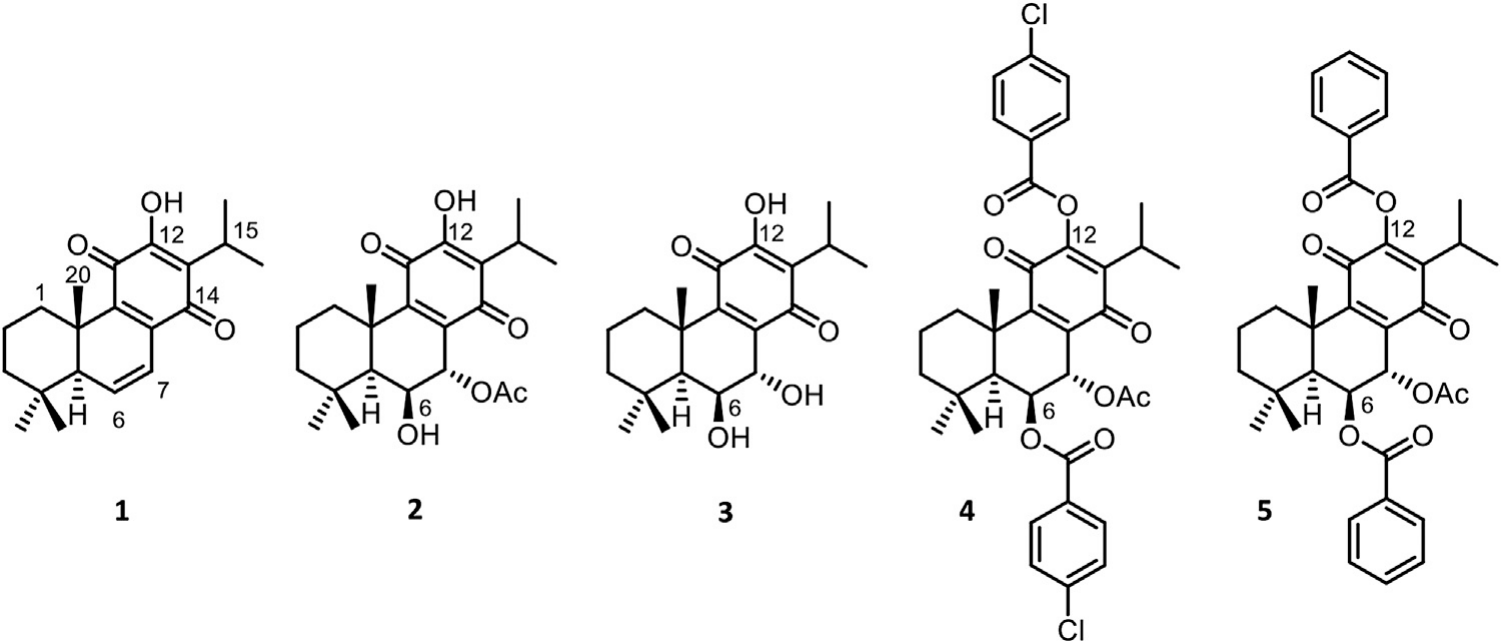
Natural and semisynthetic royleanones: 6,7-dehydroroyleanone (1) and 7α-acetoxy-6β-hydroxyroyleanone (2), 6,7-dihydroxyroyleanone (**3**), 7α-acetoxy-6β-benzoyloxy-12-*O*-(4-chloro)benzoylroyleanone (**4**) and 7α-acetoxy-6β-benzoyloxy-12-*O*-benzoylroyleanone (**5**).

The genus *Plectranthus* (Lamiaceae) is used in traditional medicine in southern Africa and it is known as a source of bioactive natural products (Lukhoba et al., 2006; Rice et al., 2011). The major classes of secondary metabolites present in these plants are diterpene quinones, coleones, and royleanones, with pharmacological activities (Bernardes et al., 2018; Rijo et al., 2013), including antiproliferative properties (Burmistrova et al., 2013; Ladeiras et al., 2016). One of those diterpenes, 6,7-dehydroroyleanone (**1**) (Figure 1), which has been reported with antioxidant, antimicrobial, and cytotoxic activities (Gazim et al., 2014; Garcia et al., 2018), is the main component of *P. madagascariensis* (Pers.) Benth essential oil (Kubínová et al., 2014). Other example is the 7α-acetoxy-6β-hydroxyroyleanone (**2**) (Figure 1), that can be isolated from extracts of *P. grandidentatus* Gürke and identified as an antimicrobial agent (Rijo et al., 2014a; Bernardes et al., 2018) with a strong inhibitory effect against five human cancer cell lines MCF-7 (breast adenocarcinoma), NCI-H460 (non-small cell lung cancer), SF-268 (CNScancer), TK-10 (renal cancer) and UACC-62 (melanoma) (Marques et al., 2002). Although the derivatization of aromatic abietane diterpenoids has been described (González, 2014), the two non-aromatic *p*-quinone abietanes, **1** and **2**, are suitable for derivatization. The analysis of the royleanone **one** chemical structure pointed to the particular acidity of the 12-hydroxyl group, due to the presence of the *p-*quinone in ring C. Alongside with the presence of this group, compound **2** possesses another free hydroxyl group at C-6, suitable for coupling different moieties.

In a previous hemi-synthetic study, the derivatives 6,7-dihydroxyroyleanone (**3**) and 7α-acetoxy-6β-benzoyloxy-12-*O*-(4-chloro)benzoylroyleanone (**4**) (Figure 1) were successfully prepared from the lead molecule **2** (Rijo, 2013). Compound **3** is a natural product isolated from *P. grandidentatus* Gürke, which can also be obtained by basic hydrolysis of compound **2** (Rijo, 2013). Furthermore, the patented diterpene 7α-acetoxy-6β-benzoyloxy-12-*O*-benzoylroyleanone (**5**) (Figure 1) was also obtained by semi-synthesis from compound **2**. The derivate **5** has shown selective modulation on Protein kinase delta isoform (PKC-δ). A key study reports that **5** strongly inhibited the proliferation of colon cancer cells by inducing a PKC-δ-dependent mitochondrial apoptotic pathway involving caspase-3 activation (Bessa et al., 2018). Besides, another study reported an important Structure-Activity Relationship (SAR) for substituted royleanone abietanes, where an electron-donating group at positions 6 and/or 7 in the abietane skeleton is required for improving cytotoxic effect. Additionally, higher cytotoxic effects were observed for substituents with log *p* values between 2 and 5 (Matias et al., 2019). Herein in this study, we report some royleanone reactivity features, which will allow us to obtain insights on the SAR and identify hit cytotoxic molecules.

## Results and Discussion

### Semisynthesis and Stability of Royleanones

**SCHEME 1:**
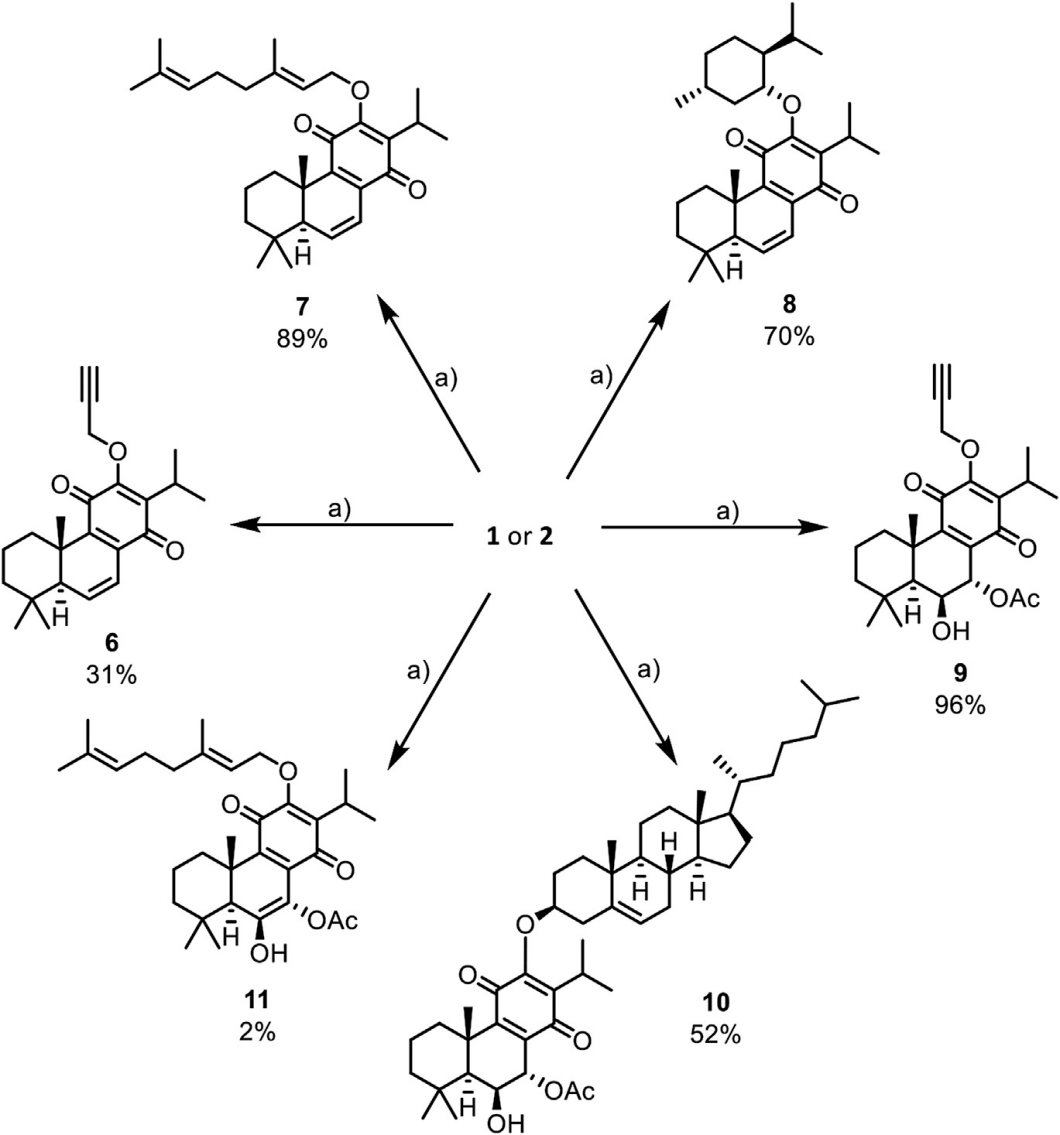
Mitsunobu reactions of natural products **1** and **2** that afford unstable derivatives: a) Triphenylphosphine (5 eq.), DIAD (5 eq.), corresponding alcohol (5 eq.), and dry THF, under argon atmosphere, derivatives **6** to **11**. *Reactional conditions described in Material and Methods (section *Reaction Procedures*) and NMR characterization available on **Supplementary Material**.

**SCHEME 2:**
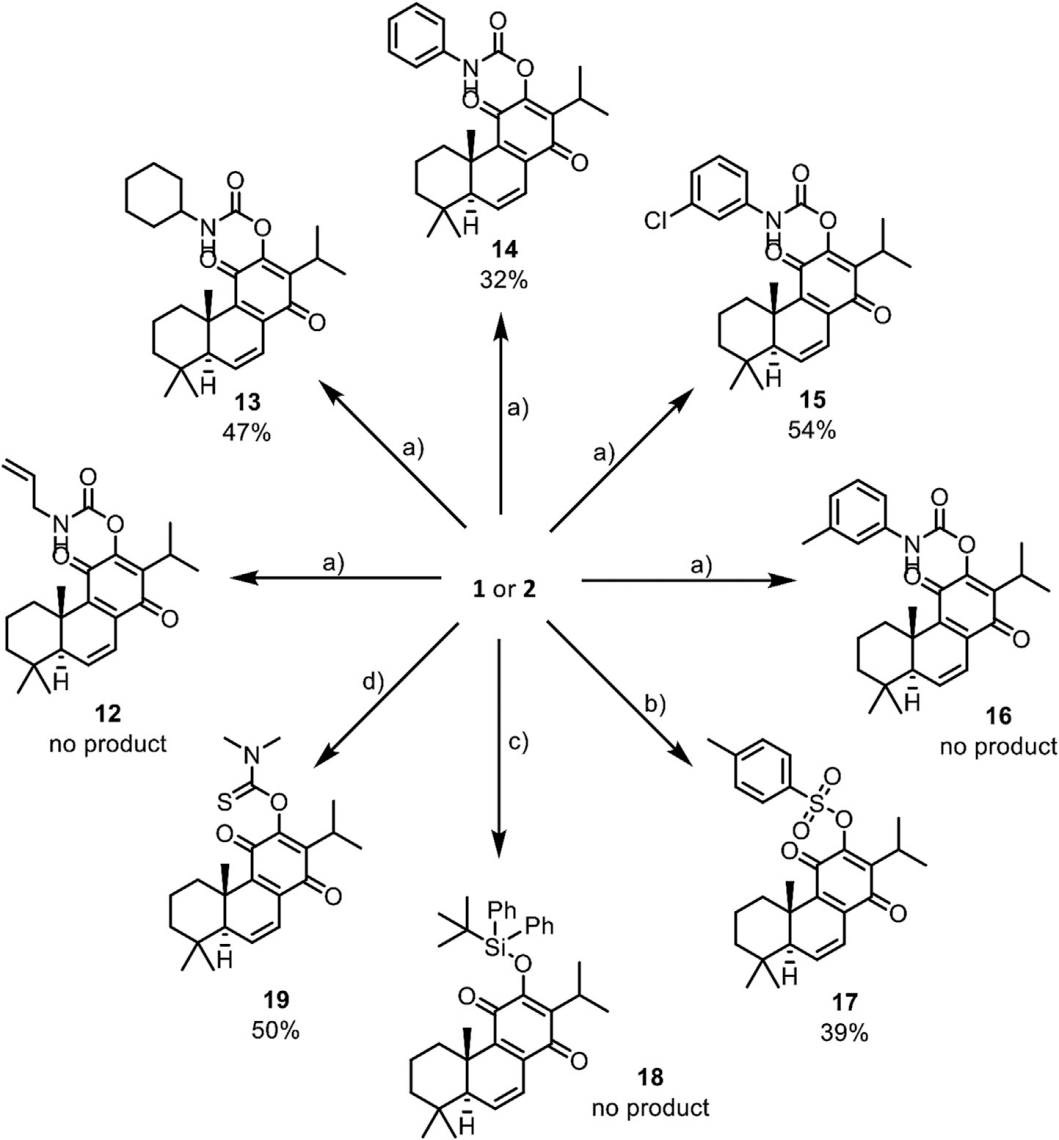
Reactions of natural products **1** and **2** that afford unstable derivatives: a) DMAP (5 eq.), corresponding isocyanate (large excess), and dry CH_2_Cl_2_, under inert conditions, expected derivatives **12** to **16**; b) triethylamine (4.5 eq.), DMAP (0.3 eq.), *p*-toluenosulfonyl chloride (3 eq.) and dry CH_2_Cl_2_, derivative **17**; d) imidazole (2 eq.), TBDPSCl (large excess) and dry CH_2_Cl_2_, expected derivative **18**; e) dimethylthiocarbamoyl chloride (1.2 eq.), NaH (1 eq), NaI (0.5 eq.) and THF, derivative **19**. *Reactional conditions described in Material and Methods (section *Reaction Procedures*) and NMR characterization available on **Supplementary Material**.

**SCHEME 3:**
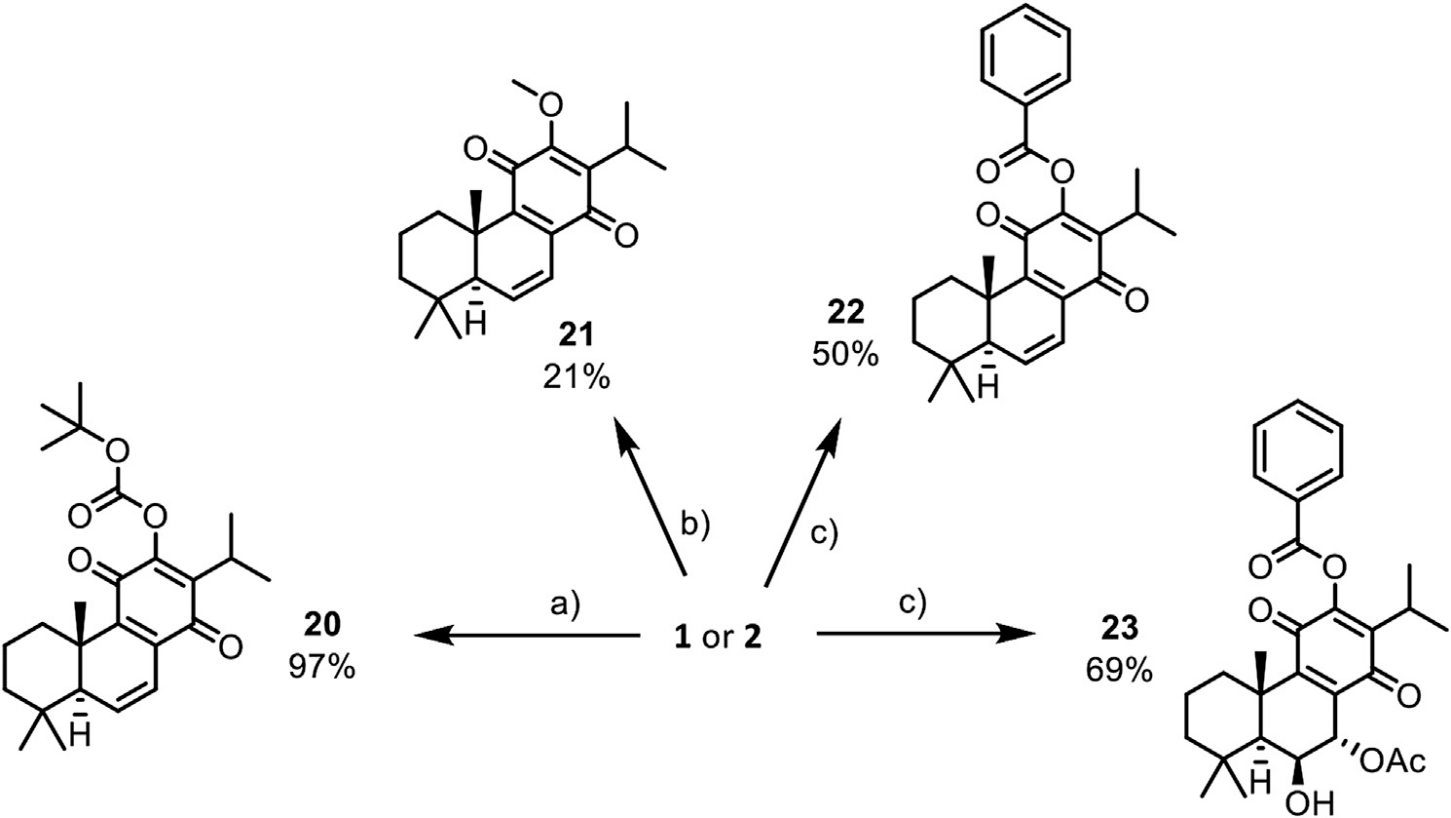
Reactions of **1** and **2** that afford stable derivatives: a) DMAP (0.5 eq.), Boc_2_O (2.2 eq.) and dry CH_2_Cl_2_, derivative **20**; b) CH_3_I (8.4 eq.), Ag_2_O (8.4 eq.) and dry CH_2_Cl_2_, derivative **21**; c) Pyridine (12 eq.), benzoyl chloride (12 eq.) and dry CH_2_Cl_2_, derivatives **22** and **23**. *Reactional conditions described in Material and Methods (section *Reaction Procedures*) and NMR characterization available on **Supplementary Material**.

In this work, the reactivity of two royleanones was explored to prepare a small library of compounds of enhanced effect on the cell viability decrease potential and anti-P-gp activity. Several hemisynthetic reactions were performed on natural compounds **1** and **2** (Figure 1). Compounds **1** and **2** were subjected to short time microwave-assisted Mitsunobu and benzoylation reactions. Additionally, molecule **1** was subjected to carbamoylation, tosylation, and introduction of TBDPS (*tert*-butyldiphenylsilyl) group. Royleanone **2** was also subjected to methylation reaction and introduction of Boc (*tert*-butyloxycarbonyl) group. The predicted structures and the isolated derivatives (**6**–**23**) are shown in **Schemes 1–3**.

Unfortunately, the obtained products have encountered stability issues: the derivatives **6** to **19** (**Scheme 1** and **2**) tend to degrade after isolation. On the other hand, the introduction of Boc group (**20**), methylation (**21**), and benzoylation (**22** and **23**) reactions (Scheme 3) were accomplished with success, affording pure products with overall good yields (97% for derivative **20**, 28% for methylated derivative **21** and 50% and 69% for benzoylated derivatives **22** and **23**, respectively).

The Mitsunobu products (**6** to **11**, Scheme 1) have displayed a high rate of decomposition, thus hampering their isolation as pure products. Several chromatographic techniques have been used, namely silica preparative TLC, silica and alumina columns as well as preparative HPLC. Despite the several purification techniques tested for the isolation of derivatives **6** to **11,** they invariably decomposed during such steps. This degradation was also observed in the carbamoylation reactions (derivatives **13** to **15**, Scheme 2), tosylation (**17**), and thiocarbamoylation (**19**). Despite the presence of allyl (**12**) and *m*-tolyl (**16**) carbamates and silyl ether (**18**) in the TLC analysis of the reaction mixtures, the compounds decomposed before purification and no characterization could be done.

The mechanism of decomposition was deduced to be the same, regardless the *O*-substituents, and we used the derivative 6,7-dehydro-12-(prop-2′-yn-1′-yloxy)-royleanone (**6**, Scheme 1), as a substrate model in further studies. The hemisynthesis of derivative **6** was repeated several times, and the isolation of the compound of interest was attempted through numerous methods. An alumina column was used for its isolation, as well as preparative TLC and semi-preparative HPLC. Nonetheless, regardless of the technique, the compound isolated was never obtained in its pure form.

In the analytical HPLC analysis of compound **6** three peaks stand out, one of which was identified as the parent compound **1** (23.31 min), using its characteristic UV spectrum as a fingerprint. This fact may indicate that compound **6** tends to decompose to a much more stable scaffold–the starting material (**1**).

**FIGURE 2:**
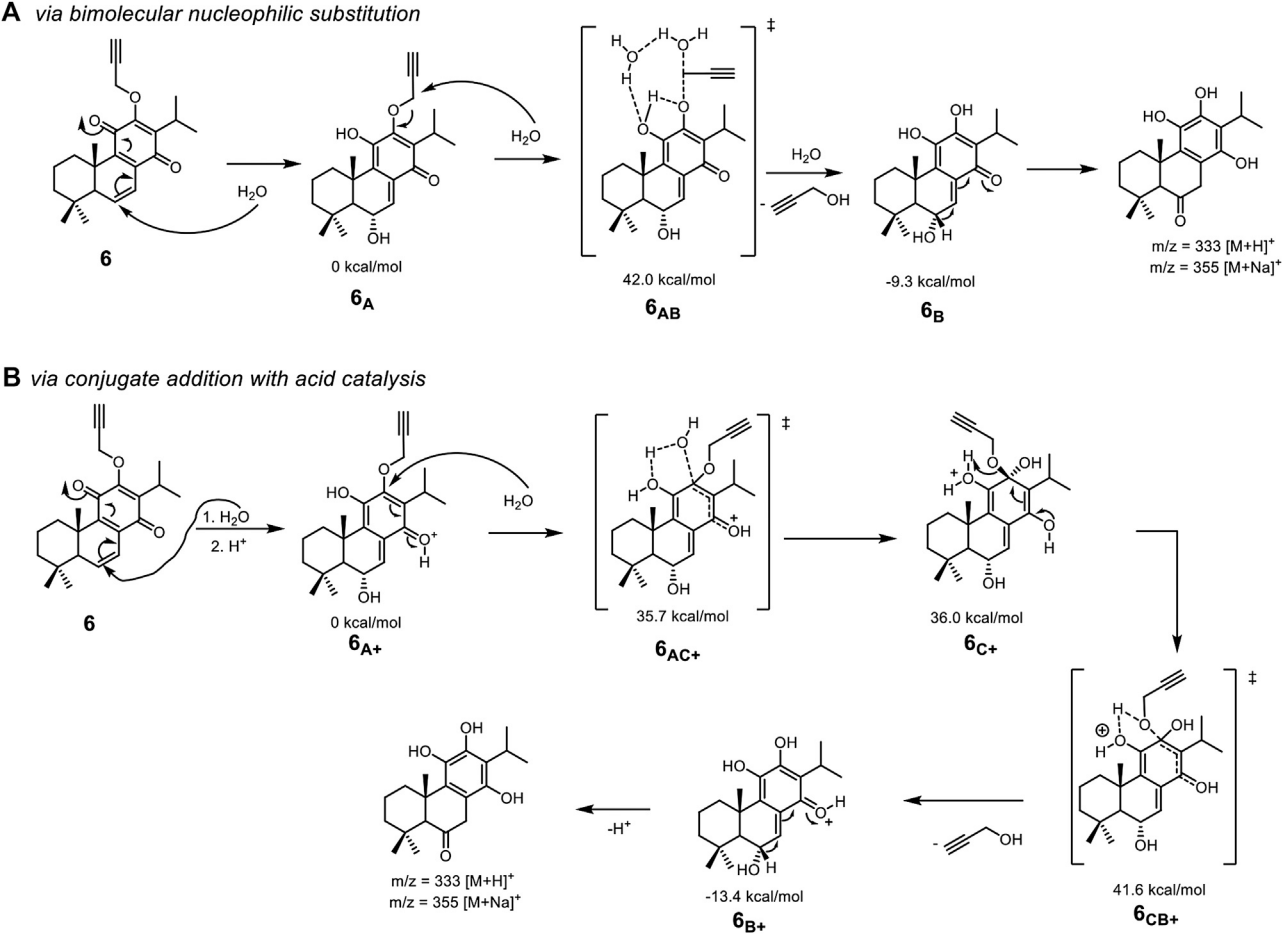
Decomposition mechanism proposal for the unstable derivatives, using derivative **6** as a working model.

In an attempt to better understand the lack of stability of the compounds, derivative **6** was subjected to LC-MS analysis. The ESI positive mass spectra indicated a mass of m/z 355 [M + Na]^+^. Although it was not possible to identify the moiety or cleavage pattern responsible for the result, some decomposition mechanisms based on the presence of the mentioned fragment were considered (Figure 2).

### Decomposition Mechanism of Unstable Derivatives

The decomposition of derivative **6** was studied by Density Functional Theory (Parr and Yang, 1989) to elaborate a mechanism and facilitate the future preparation of more stable derivatives (Figure 2). Different mechanisms for decomposition have been suggested based on computational calculations. Nucleophilic substitution at a more reactive side chain seems the preferential route, while an acid-catalyzed conjugate addition should operate in the case of less electrophilic side chains. The decomposition is likely to start with a nucleophilic attack by water on position 6 to provide **6**_**A**_, being followed by a bimolecular nucleophilic substitution by water on the propargylic position, through calculated **6**_**AB**_, as shown in Figure 2A). This second step was determined to have an energy barrier (ΔG^‡^) of 42.0 kcal/mol and, upon the establishment of hydrogen bonds network with water molecules. Notwithstanding the high energy barrier, the process is energetically favorable with the intermediate **6**_**B**_ being 9.3 kcal/mol more stable than **6**_**A**_. The 1,2-hydride migration in **6**_**B**_ delivers the aromatized molecule identified in HRMS.

The attack of water at position 12 of **6**_**A**_ in a conjugate addition fashion, was considered but determined highly unlikely due to an energy barrier of 55.6 kcal/mol. However, this energy barrier lowers significantly when in the presence of acid catalysis (Figure 2B). In that case, tetrahedral intermediate **6**_**C+**_ was identified in the computational calculations, with the overall energy barrier of the release of propargyl alcohol being 41.6 kcal/mol. This alternative mechanism, in which a proton source is required, is likely to be more relevant in decomposing derivatives that miss the electrophilic position prone for an SN2 reaction. This being the case for most of the unstable products.

### Effects of Royleanones in Multidrug Resistance Mechanisms of Cancer Cells

The P-gp inhibition potential of all stable derivatives obtained, derivatives **20** to **23**, was investigated. Moreover, derivative **4**, previously prepared (Rijo, 2013), was also assessed. Non-small cell lung carcinoma is particularly hard to treat due to its highly resistant and metastatic profile. Therefore, NCI-H460 and its corresponding MDR cell line NCI-H460/R with the overexpression of P-gp was a suitable model for testing the anticancer effect and P-gp inhibitory effect of our compounds. MRC-5 was selected as a normal cell line due to its bronchial epithelial origin. The effect of derivatives **20** to **23** was illustrated according to the fluorescence activity ratio (FAR) and sensitivity index (SI) (Table 1). Based on the FAR (values above 1.50 indicate P-gp inhibition) and SI (values above 20 account for P-gp inhibition), we could see that only derivatives **4** and **23** have the ability to inhibit P-gp activity, with FAR values of 1.71 and 2.10, respectively, and SI of 21.60 and 26.50, respectively. Additionally, derivative **23** has shown a comparable inhibitory potential to the well-known P-gp inhibitor, Dexverapamil (FAR 2.13 and SI 26.90) (Isca et al., 2020). A recent study used molecular docking and molecular dynamics to explore the interaction of derivatives **4** and **23** with P-gp and suggested that the presence of aromatic moieties increases the binding affinity of royleanone derivatives toward P-gp (Isca et al., 2020). On the other hand, derivative **22** is also a benzoylated derivative, nonetheless, it does not show the ability to inhibit P-gp activity. The difference between derivatives **4**, **23**, and **22** is that they are obtained from different natural products. Namely, derivative **4** and **23** are prepared from royleanone **2,** and derivative **22** is obtained from royleanone **1**. It means that derivatives **4** and **23** displayed a hydroxyl group in position 6 and an acetoxy group in position 7, while derivative **22,** displayed a double-bound (C=C) in these 6 and 7 positions. This suggests that the substituents in position 6 (-OH) and 7 (-OAc) can also contribute to P-gp interaction. Further studies should be conducted to assess this hypothesis.

**TABLE 1:** P-gp inhibition by derivatives 4, 20, 21, 22, and 23 in the human NSCLC MDR cancer cell line.

Treatments	MFI^a^	FAR^b^	SI^c^
NCI-H460 control	134.10		
NCI-H460/R control	16.96		12.65
DexVER	36.07	2.13	26.90
4^d^	28.97	1.71	21.60
20	23.71	1.40	17.68
21	19.48	1.15	14.53
22	18.25	1.08	13.61
23^d^	35.54	2.10	26.50

a The measured mean fluorescence intensity (MFI) was used for the calculation of the fluorescence activity ratio (FAR).

b via the following equation: FAR = MFI_MDR treated_/MFI_MDR control_. FAR values above 1.50 indicate P-gp inhibition.

c The sensitivity index (SI) was calculated on the basis of the measured mean fluorescence intensity (MFI) expressed via the following equation: SI = (MFI_MDR treated_ * 100)/MFI_sensitive control_. SI values above 20 account for P-gp inhibition.

d Results published in Isca et al. (2020).

Sensitive cancer cell line and its MDR counterpart used in the study: non-small cell lung carcinoma-NSCLC (NCI-H460 and NCI-H460/R).

DexVER was applied at the same concentration (20 µM) as tested derivatives.

Derivatives **4** and **23** showed to be promising candidates for P-gp inhibition, nonetheless, due to the small amount of compound **23** available, we choose the royleanone **4** for further studies. Accordingly, the effect of compound **4** was investigated in NCI-H460, NCI-H460/R cell lines, and normal embryonal bronchial epithelial cells MRC-5. Royleanone **4** showed high toxicity in all cell lines tested, with IC_50_ of 1.9 ± 0.4 µM for NCI-H460, 2.2 ± 0.4 µM for NCI-H460/R, and 2.0 ± 0.3 µM for MRC-5 cell lines. In previous studies, Garcia et al. (2018) and Matias et al. (2019) established the toxicity of the natural diterpenes **1**, **2**, and **3** in the same cell lines. Royleanone **2** is more efficient than **1** and **3**, with IC_50_ of 2.7 µM for NCI-H460, 3.1 µM for NCI-H460/R, and 8.6 µM for MRC-5 cell lines (Garcia et al., 2018; Matias et al., 2019). According to these results, the derivatization of royleanone **2** into derivative **4** lead to a decrease in cell viability in all cell lines tested. However, compounds **1**, **2,** and **three** were selective toward cancer cells (Garcia et al., 2018; Matias et al., 2019), while derivative **4** was equally active against normal cells.

In general, P-gp inhibitors can block drug binding sites either competitively, non-competitively, or allosterically. Many inhibitors, namely, verapamil, cyclosporin A, *trans*-flupenthixol, among others, are themselves transported by P-gp (Amin, 2013). On the contrary, royleanones **1–4** showed the same efficacy in sensitive and MDR cancer cells implying that they could not be P-gp substrates. Moreover, Isca et al. (2020) based on docking simulations also suggest that derivatives **4** and **23** act as non-competitive efflux inhibitors.

**FIGURE 3:**
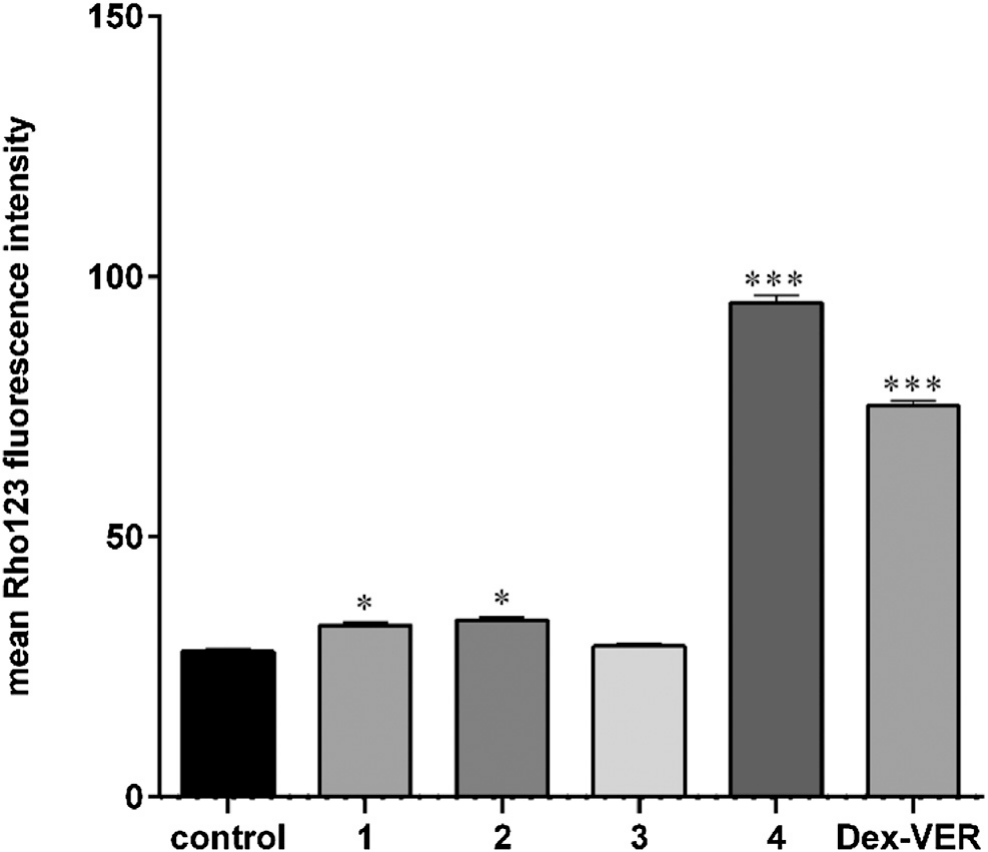
Royleanone derivatives increase the Rhodamine 123 accumulation implying anti-P-gp activity. Experiments were performed in triplicates (n = 3). Significant difference compared to control: * p < 0.05, *** p < 0.001.

The P-gp inhibiting effects of compounds **1** to **4** were additionally assessed through a Rhodamine 123 (Rho123) accumulation assay (Figure 3). The obtained results indicate that derivative **4** has comparable inhibitory potential to Dexverapamil (Figure 3). Dexverapamil belongs to the second generation of P-gp inhibitors, known as a competitive inhibitor (Robey et al., 2018). In our experiments with Rho123, Dexverapamil competes with Rho123 for binding P-gp and thus increases the Rho123 accumulation. Recent publications imply that verapamil (first-generation inhibitor) can increase the ATPase activity of P-gp and thus by exhausting the ATP suppress P-gp function (Lee et al., 2019). It is well known that some substrates of P-gp can exert an inhibitory effect on P-gp if they are applied in higher concentrations (Durmus et al., 2015). On the other side, there are substrates of P-gp such as doxorubicin which cannot inhibit P-gp. Quite opposite, doxorubicin induces the expression of P-gp (Wu et al., 2016). Accordingly, we also evaluated the ability of **4** to sensitize resistant NCI-H460/R cells to doxorubicin (Table 2). Results showed that derivative **4** was able to sensitize MDR cells to doxorubicin. All three concentrations of compound **4** used to reverse the doxorubicin resistance achieved similar efficacy. Importantly, sub-IC_50_ concentrations (0.5 and 1 μM) can reverse doxorubicin resistance. Therefore, derivative **4** can be considered as a new P-gp inhibitor useful in combination with classic chemotherapeutics.

**TABLE 2:** Derivative 4 sensitizes the NCI-H460/R cell line to doxorubicin.

Combined treatments	IC_50_ for DOX (µM)	Relative reversal factor
DOX	2.774 ± 0.025	—
4 (0.5 µM) + DOX	0.823 ± 0.016	3.37
4 (1.0 µM) + DOX	0.594 ± 0.017	4.67
4 (2.0 µM) + DOX	0.608 ± 0.020	4.56

DOX concentrations used in the experiments: 0.1, 0.25, 0.5, 1 and 2.5 μM.

## Conclusions

In this work, the reactivity of two natural royleanones was explored to obtain a small library of new P-gp modulators. Several hemisynthetic reactions were performed and successful results were obtained when performing methylation and benzoylation, and introduction of Boc group, affording compounds **20** to **23** as pure with overall good yields.

P-gp inhibition potential of the stable derivatives (**20**–**23**) was evaluated. Previously prepared diterpene **4**, has also been tested. From the tested derivatives, compounds **4** and **23** have significant P-gp inhibitory potential.

Regarding stability and P-gp inhibition potential, results suggest that the formation of benzoyl esters is a more convenient approach for future derivatives with enhanced cytotoxicity. Furthermore, this study suggests that the moieties in positions 6 and 7 of royleanones are also important for interaction with the P-pg. Further studies are needed to disclosure this hypothesis.

Additional studies with royleanone **4**, indicate an increase of anti-P-gp potential in comparison to the natural diterpenes **1**, **2**, and **3**, similar to Dexverapamil inhibitory potential. Moreover, derivative **4**, showed the ability to sensitize the resistant NCI-H460/R cells to doxorubicin. This diterpene could be considered as a novel P-gp inhibitor useful in combination with classic chemotherapeutics.

## Materials and Methods

### Plant Material

The plant material, *P. madagascariensis* (Pers.) Benth. and *P. grandidentatus* Gürke were cultivated in Parque Botânico da Tapada da Ajuda (Instituto Superior Agrário, Lisbon, Portugal) from cuttings obtained from the Kirstenbosch National Botanical Garden (Cape Town, South Africa). Voucher specimens were deposited in Herbarium João de Carvalho e Vasconcellos (ISA). The plant name has been checked with http://www.theplantlist.org (The Plant List. Version 1.1, 2013). The extraction and isolation process of **1** and **2** were performed according to Garcia et al. (2018) and Bernardes et al. (2018), respectively.

### Reaction Procedures

The Mitsunobu reactions were carried out with microwave irradiation, according to a previous report (Buonomo and Aldrich, 2015): **1** (10 mg, 0.032 mmol) or **2** (10 mg, 0.026 mmol), corresponding alcohol (5 eq.), triphenylphosphine (5 eq.) and DIAD (5 eq.) in 4.5 ml dry THF, were irradiated with microwaves at 300 W and 60 °C for 45 min under argon atmosphere. Conditions for Carbamoylation: A mixture of **1** (20 mg, 0.064 mmol), DMAP (5 eq.) and excess of the corresponding isocyanate, in 0.5 ml dry CH_2_Cl_2_, were stirred at room temperature under inert conditions until consumption of the starting material as judged from TLC. Tosylation Conditions: A mixture of **1** (15 mg, 0.048 mmol), triethylamine (4.5 eq.), DMAP (0.3 eq.) and *p*-toluenosulfonyl chloride (3 eq.), in 0.5 ml dry CH_2_Cl_2_ were stirred until consumption of the starting material as judged from TLC. Introduction of the TBDPS group: A mixture of **1** (15 mg, 0.048 mmol), imidazole (2 eq.) and excess of *tert*-butyldiphenylchlorosilane in 1 ml dry CH_2_Cl_2_ was stirred at room temperature Thiocarbamoyl: **1** (15 mg, 0.048 mmol) was added to a suspension of sodium hydride (1 eq.) in 0.4 ml THF, followed by sodium iodide (0.5 eq.) and dimethylthiocarbamoyl chloride (1.2 eq.). The mixture was left stirring at room temperature until consumption of the starting material. Introduction of Boc group: A mixture of **1** (15 mg, 0.048 mmol), DMAP (0.5 eq.) and Boc_2_O (2.2 eq.) in 0.5 ml of dry CH_2_Cl_2_ was left stirring at room temperature. Methylation: A mixture of **1** (15 mg, 0.048 mmol) (1 eq.), methyl iodide (8.4 eq.) and silver oxide (8.4 eq.), in 0.5 ml of dry CH_2_Cl_2_ was left stirring at room temperature. Benzoylation: A mixture of **1** (15 mg, 0.048 mmol) or **2** (10 mg, 0.026 mmol), pyridine (12 eq.) and benzoyl chloride (12 eq.), in 2 ml dry CH_2_Cl_2_ was left stirring at room temperature until complete consumption of the starting material as judged by TLC.

### Semi-preparative HPLC-Diode Array Detector Analysis

The analytical method was carried out in an Agilent Technologies 1200 Infinity Series system with a diode array detector (DAD), equipped with a Zorbax® XDB-C18 (250 × 4.0 mm i.d., 5  μm) column, from Merck and ChemStation Software. The sample was injected in acetone, 10 mg/ml. Each injection was analyzed with a gradient elution mixture composed of solution A (methanol), solution B (acetonitrile), and solution C (0.3% trichloroacetic acid in water) was used as follows: 0 min, 15% A, 5% B, and 80% C; 20 min, 70% A, 30% B and 0% C; 25 min, 70% A, 30% B and 0% C; and 28 min, 15% A, 5% B and 80% C. The flow rate was set at 1 ml/min and 20 μL of the sample were injected.

### Chemical Stability Evaluation by LC-MS

LC-MS/MS analysis was performed using a Zorbax Eclipse XBD-C18, 4.6 × 250 mm (5 μm) and the mobile phase consisted of 0.5% formic acid in Milli-Q water (eluent A) and acetonitrile + 0.5% formic acid (eluent B). A flow rate of 0.3 ml/min was used, with the following gradient program: 0–30 min from 70 to 5% A, 30–45 min at 5% A, 45–65 min 70% A.

### Cells and Cell Culture

Non-small cell lung carcinoma cell line NCI-H460 was purchased from the American Type Culture Collection, Rockville, MD. NCI-H460/R cells were selected originally from NCI-H460 cells and cultured in a medium containing 100 nM doxorubicin (Pesic et al., 2006). Cell lines were subcultured at 72 h intervals using 0.25% trypsin/EDTA and seeded into a fresh medium at the following densities: 8,000 cells/cm^2^ for NCI-H460 and 16,000 cells/cm^2^ for NCI-H460/R.

### MTT Test

MTT assay is based on the reduction of 3-(4, 5-dimethyl-2-thizolyl)-2,5-diphenyl-2H-tetrazolium bromide into formazan dye by active mitochondria of living cells. Cells grown in 25 cm^2^ tissue flasks were trypsinized, seeded into flatbottomed 96-well tissue culture plates (2,000 cells/well), and incubated overnight in 100 µL of appropriate medium. After 24 h, the cells were treated with compounds **1** to **4** (1–25 µM) and incubated for 72 h in complete medium. The combined effects of **4** simultaneously applied with doxorubicin were also studied. In simultaneous treatments, three concentrations of **4** (0.5, 1, and 2 µM) were combined with five concentrations of doxorubicin (0.1, 0.25, 0.5, 1, and 2.5 µM). After 72 h, 100 µL of MTT solution (1 mg/ml) was added to each well, and plates were incubated at 37°C for 4 h. Formazan product was dissolved in 200 ml dimethyl sulfoxide. The absorbance of the obtained dye was measured at 540 nm using an automatic microplate reader (LKB 5060–006 Micro Plate Reader, LKB, Vienna, Austria). Half maximal inhibitory concentration (IC_50_ value) was defined as the concentration of the drug that inhibited cell growth by 50% and calculated by non-linear regression analysis using GraphPad Prism6 software.

### Rhodamine 123 Flow Cytometry Assay

Rhodamine 123 accumulation was analyzed by flow cytometry utilizing the ability of Rhodamine 123 to emit fluorescence. The intensity of the fluorescence is proportional to Rhodamine 123 accumulation. Studies were carried out with Dexverapamil and compounds **1** to **4**. NCI-H460 and NCIH460/R cells were grown to 80% confluence in 75 cm^2^ flasks, trypsinized, and resuspended in 10 ml centrifuge tubes in a Rhodamine 123-containing medium. The cells were treated with diterpenes and Dexverapamil (5 and 20 μM) and incubated at 37°C in 5% CO_2_ for 30 min. At the end of the incubation period, the cells were pelleted by centrifugation, washed with PBS, and placed in cold PBS. The samples were kept on ice in dark until the analysis of the CyFlow Space Partec flow-cytometer (Sysmex Partec GmbH, Germany). The fluorescence of Rhodamine 123 was assessed on the FL1 channel. A minimum of 20,000 events was assayed for each sample and the obtained results were analyzed using Summit Dako Software.

### Density Functional Theory

All calculations were performed using the Gaussian 16 software package (Frisch et al., 2016), without symmetry constraints. The PBE1PBE functional was employed in the geometry optimizations. That functional uses a hybrid generalized gradient approximation (GGA), including a 25% mixture of Hartree-Fock (Hehre et al., 1986) exchange with DFT (Parr and Yang, 1989) exchange-correlation, given by Perdew, Burke, and Ernzerhof functional (PBE) (Perdew, 1986; Perdew et al., 1997). The optimized geometries were obtained with a standard 6–31G(d,p) (Ditchfield et al., 1971; Hehre et al., 1972; Hariharan and Pople, 1974; Gordon, 1980) basis set.

Transition state optimizations were performed with the Synchronous Transit-Guided Quasi-Newton Method (STQN) developed by Peng and Bernhard Schlegel (1993) and Peng et al. (1996). Frequency calculations were performed to confirm the nature of the stationary points, yielding one imaginary frequency for the transition states and none for the minima. Each transition state was further confirmed by following its vibrational mode downhill on both sides and obtaining the minima presented on the energy profile.

## Data Availability Statement

The raw data supporting the conclusions of this manuscript will be made available by the authors, without undue reservation, to any qualified researcher.

## Author Contributions

The manuscript was written through the contributions of all authors.

## Funding

Support for this work was provided by FCT through UIDP/04567/2020, UIDB/04567/2020, and Ph.D. grant SFRH/BD/137671/2018. CSC-IT center for Science Ltd., Finland is acknowledged for the computational resources’ allocation. The Academy of Finland is acknowledged for the financial support to N.R.C (Decisions No. 326487 and 326486).

## Conflict of Interest

The authors declare that the research was conducted in the absence of any commercial or financial relationships that could be construed as a potential conflict of interest.
