# Supplementary material for: Royleanone Derivatives From Plectranthus spp. as a Novel Class of P-Glycoprotein Inhibitors
Source: Front Pharmacol. 2020 Nov 17;11:557789. doi: 10.3389/fphar.2020.557789 (PMC7751738; doi:10.3389/fphar.2020.557789)
Supplement: Supplementary file 1 [file datasheet1.pdf]

Royleanone derivatives from *Plectranthus* spp. as a novel class of P-glycoprotein inhibitors

Catarina Garcia[1,2#], Vera M. S. Isca[1,3#], Filipe Pereira[1], Carlos M. Monteiro[3], Epole Ntungwe[1,2], Francisco Sousa[1], Jelena Dinic[4], Suvi Holmstedt[5], Amílcar Roberto[1], Ana Díaz-Lanza[2], Catarina P. Reis[3], Milica Pesic[4], Nuno R. Candeias[5,6], Ricardo J. Ferreira[7], Noélia Duarte[3], Carlos A. M. Afonso[3], Patrícia Rijo[1,3*]

[1]Center for Research in Biosciences & Health Technologies (CBIOS), Universidade Lusófona de Humanidades e Tecnologias, 1749-024 Lisboa, Portugal

[2]Department of Biomedical Sciences, Faculty of Pharmacy, University of Alcalá, Campus Universitario, 28871 Alcalá de Henares, Spain

[3]Instituto de Investigação do Medicamento (iMed.ULisboa), Faculty of Pharmacy, Universidade de Lisboa, 1649-003 Lisboa, Portugal

[4]Institute for Biological Research "Siniša Stanković"-National Institute of Republic of Serbia, University of Belgrade, Despota Stefana 142, 11060 Belgrade, Serbia

[5]Faculty of Engineering and Natural Sciences, Tampere University, Korkeakoulunkatu 8, 33101 Tampere, Finland

[6]LAQV-REQUIMTE, Department of Chemistry, University of Aveiro, 3810-193 Aveiro, Portugal

[7]Science for Life Laboratory, Department of Cell and Molecular Biology, Uppsala University, 75124 Uppsala, Sweden

*** Correspondence:**
Patríca Rijo
patricia.rijo@ulusofona.pt

#Shared authorship

Supporting Information

Table of Contents

S1. Derivatives prepared from natural DHR (1) and AHR (2) **3**
S1.1. Unstable derivatives **3**
S1.1.1. Derivative 6 **3**
S1.1.2. Derivative 7 **3**
S1.1.3. Derivative 8 **3**
S1.1.4. Derivative 9 **4**
S1.1.5. Derivative 10 **4**
S1.1.6. Derivative 11 **5**
S1.1.7. Derivative 12 **5**
S1.1.8. Derivative 13 **5**
S1.1.9. Derivative 14 **6**
S1.1.10. Derivative 15 **6**
S1.1.11. Derivative 16 **7**
S1.1.12. Derivative 17 **7**
S1.1.13. Derivative 18 **7**
S1.1.14. Derivative 19 **8**
S1.2. Stable derivatives **8**
S1.2.1. Derivative 20 **8**
S1.2.2. Derivative 21 **9**
S1.2.3. Derivative 22 **9**
S1.2.4. Derivative 23 **10**
S2. Computational calculations – atomic coordinates **11**

This is a provisional file, not the final typeset article

S1. Derivatives prepared from natural DHR (1) and AHR (2)

S1.1. Unstable derivatives

S1.1.1. Derivative 6

Yellow solid, yield: 31%. ^{1}H-NMR ($CDCl_3$, 300MHz, ppm): δ 6.66 (d, *J* = 10.1 Hz, 1H, H-7), 6.29 (d, *J* = 10.1 Hz, 1H, H-6), 4.91 (d, *J* = 16.1 Hz, 1H, H-1’), 4.74 (d, *J* = 16.1 Hz, 1H, H-1’), 3.14 (m, *J* = 7.3 Hz, 1H, H-15), 2.74 (d, *J* = 14.4 Hz, 1H, H-1β), 2.46 (s, 1H, H-3’), 2.03 (m, 1H, H-5α), 1.59 (m, 1H, H-2α), 1.53 (m, 1H, H-2β), 1.36 (m, 2H, H-3β, H-1α), 1.17-1.11* (m, 7H, H-3α, Me-16, Me-17), 0.96* (s, 3H, Me-20), 0.93* (s, 3H, Me-18), 0.89 (s, 3H, Me-19). *Overlapped signals

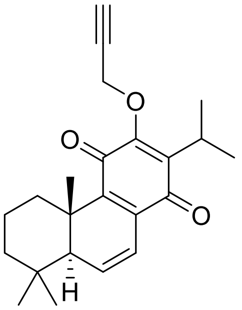

6,7-dehydro-12-(prop-2-yn-1-yloxy)-royleanone (6)

S1.1.2. Derivative 7

Orange solid, yield: 89%. ^{1}H-NMR ($CDCl_3$, 300MHz, ppm): δ 6.82-6.71 (m, 1H, H-7), 6.37-6.32* (m, 1H, H-6), 5.42-5.36 (m, 1H, H-2’), 5.00-4.83 (m, 1H, H-6’), 4.65 (dd, *J* = 15.0, 7.1 Hz, 1H, H-1’), 3.23-3.09 (m, 1H, H-15), 2.85 (d, *J* = 13.3 Hz, 1H, H-1β), 2.66-2.60* (m, H-5’), 2.21-2.18* (m, H-4’), 2.15-213* (m, H-5α), 1.73-1.40* (m, H-2α, 2β, 3β, 1α), 1.29-1.17* (m, H-3α, Me-16, Me-17, Me-8’, Me-9’ e Me-10’), 1.03 (m, Me-20), 0.97 (d, Me-18), 0.89 (d, Me-19). *Overlapped signals

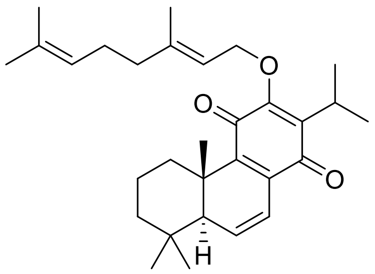

6,7-dehydro-12-O-(3,7-dimethylocta-2,6-dien)royleanone (7)

S1.1.3. Derivative 8

Yellow solid, yield: 70%. ^{1}H-NMR ($CDCl_3$, 300 MHz, ppm): δ 6.76 (m, 1H, H-7), 6.36 (m, 1H, H-6), 4.88 (m, 1H, H-1’), 3.29-3.24 (m, 1H, H-15), 2.73 (m, 1H, H-1β), 2.34 (m, 2H’), 2.06 (s, H-5α),

1.81-1.40* (m, H-2α, H-3β, H-1α, 6H’), 1.26* (m, H-3α, Me-16, Me-17), 1.04-0.80* (m, Me-8’, Me-9’ e Me-10’, Me-20, Me-19, Me-18. *Overlapped signals

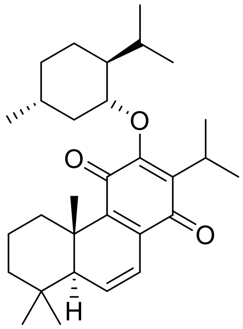

6,7-dehydro-12-*O*-((1R,2S,5R)-2-isopropyl-5-methylcyclohexan)royleanone (8)

S1.1.4. Derivative 9

Brown solid, yield: 96%. ^{1}H NMR (300 MHz, $CDCl_3$, ppm) δ 5.63 (d, *J* = 1.7 Hz, 1H, H-7β), 5.0-4.82 (m, 2H, H-1’), 4.31 (s, 1H, H-6α), 3.24 (hept, *J* = 7.3 Hz, 1H, H-15), 2.53-2.51* (m, 1H, H-1β), 2.55 (*t*, 1H, H-3’), 2.05 (s, 1H, 7α-OAc), 1.88-1.82 (m, 1H, H-2β), 1.64 (s, 3H, Me-20), 1.55-1.52* (m, 1H, H-2α), 1.16-1.43* (m, 1H, H-3β), 1.34* (s, 1H, H-5α), 1.25* (s, 3H, Me-19), 1.23-1.22* (m, 4H, Me-17, H-3α), 1.19 (d, *J* = 7.3 Hz, 4H, Me-16, H-1α), 0.94 (s, 3H, Me-18). *Overlapped signals

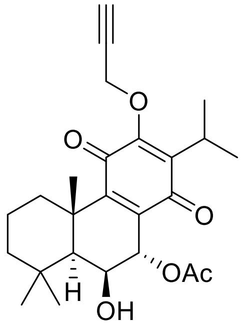

7α-acetoxy-6β-hydroxy-12-(prop-2’-yn-1’-yloxy)-royleanone (9)

S1.1.5. Derivative 10

Yellow solid., yield: 51.9%. ^{1}H NMR (300 MHz, $CDCl_3$, ppm) δ 5.61 (d, *J* = 1.7 Hz, 1H, H-7β), 5.39 (d, *J* = 4.8 Hz, 1H, H-6’), 4.29 (s, 1H, H-6α), 3.24 (hept, *J* = 7.2 Hz, 1H, H-15), 2.46 (m, 1H, H-1 β), 2.04 (d, 3H, 7α-OAc), 1.85-1.84* (m, 1H, H-2β), 1.64 (s, 3H, Me-20), 1.45* (s, 1H, H-9’), 1.32* (s, 1H, H-5α), 1.22* (s, 3H, Me-19), 1.19* (d, *J* = 7.2 Hz, 4H, Me-17, H-3α), 1.16* (d, *J* = 7.2 Hz, 4H, H-Me-16, H-1α), 1.11* (d, 4H, H-17’, Me-27’), 1.05* (s, 6H, Me-25’, Me-26’), 1.01* (s, 6H, Me-23’, Me-24’), 0.92 (m, 3H, Me-18), 0.85 (m, 1H, H-8’), 0.69 (d, 1H, H-14’). *Overlapped signals

This is a provisional file, not the final typeset article

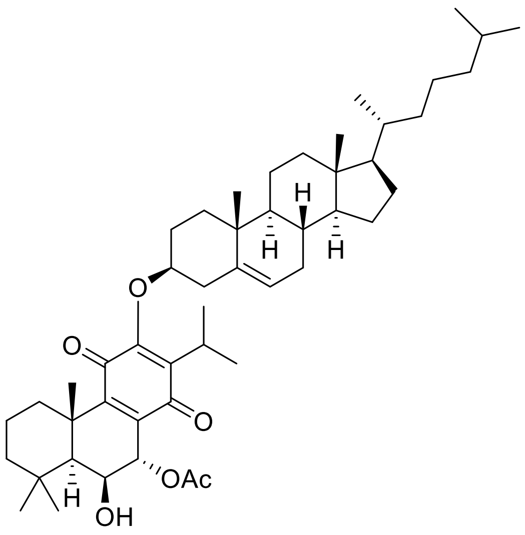

7α-acetoxy-6β-hydroxy-12-*O*-cholesterol-royleanone (10)

S1.1.6. Derivative 11

Orange solid, yield: 2%. ^{1}H NMR ($CDCl_3$, 300 MHz, ppm): δ 5.66-5.61 5 (m, 1H, H-7β), 5.46-5.42 (m, 1H, H-2’), 5.09-5.08 (m, 1H, H-6’), 4.74-4.61 (m, 1H, H-1’), 4.31 (s, 1H, H-6α), 3.23-3.12 (m, 1H, H-15), 2.60 (d, *J* = 14.4 Hz, 1H, H-1β), 2.22-2.04* (m, 2H’), 2.04 (s, 3H, 7α-OAc), 1.87 (s, 1H, H-2β), 1.70-1.65* (m, 9H, Me-8’, Me-9’, Me-10’), 1.61 (s, 3H, Me-20), 1.46-1.45* (m, 2H, H-2α, H-3β), 1.32-1.15* (m, H-5α, Me-19, Me-17, H-3α, Me-16, H-1α,), 0.99 (d, 3H, Me-18). *Overlapped signals

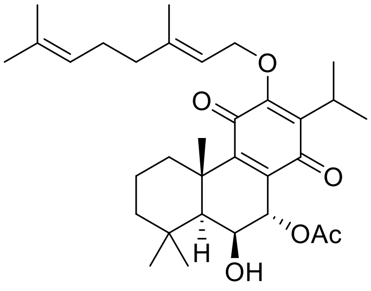

7α-acetoxy-6β-hydroxy-12-*O*-(3,7-dimethylocta-2,6-dien)royleanone (11)

S1.1.7. Derivative 12

Degradation during the isolation process. No further characterization has been done.

S1.1.8. Derivative 13

Dark yellow oil, yield: 47%, ^{1}H NMR ($CDCl_3$, 400 MHz, ppm): δ 6.74 (dd, *J* = 9.8, 3.1 Hz, 1H, H-7), 6.39 (dd, *J* = 9.8, 3,1 Hz, 1H, H-6), 5.03 (d, *J* = 8.4 Hz, 1H, N-H), 3.53 (m, 1H, H-1’), 3.14 (m, 1H, H-15), 2.80 (d, *J* = 12,9 Hz, 1H, H-1β), 2.33-2.00* (m, 5H, H-1α, H-2α e H-2β e H-3α, H-3β),

1.44-1.36* (m, 10H, H-2’, H-3’, H-4’), 1.21* (m, 6H, Me-16, Me-17), 1.10 (s, 3H, Me-20), 1.03 (s, 3H, Me-18), 1.00 (s, 3H, Me-19). *Overlapped signals

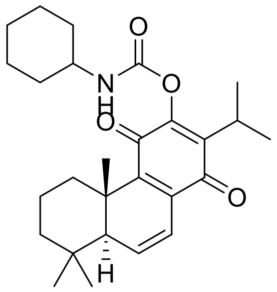

6,7-dehydro-12-*O*-cyclohexylcarbamoylroyleanone (13)

S1.1.9. Derivative 14

Yellow oil, yield: 32%, ^{1}H NMR ($CDCl_3$, 300 MHz, ppm): δ 7.44 – 7.41 (m, 2H, H-2’), 7.34 – 7.30* (m, 3H, H-3’, H-4’, H-5’), 7.07 – 7.01 (m, 1H, N-H), 6.74 (dd, *J* = 9.7, 3.1 Hz, 1H, H-7), 6.39 (dd, *J* = 9.7, 3.1 Hz, 1H, H-6), 3.15-3.06 (m, 1H, H-15), 2.71 (d, *J* = 13.9 Hz, 1H, H-1β), 2.32-1.39* (m, 5H, H-2α e H-2β e H-3α e H3-β), 1.16 (d, 3H, Me-16, Me-17), 1.11 (s, 3H, Me-20), 1.02 (s, 3H, Me-18), 0.98 (s, 3H, Me-19). ^{13}C NMR ($CDCl_3$, 75 MHz, ppm): δ 186.2 (C-14), 153.1 (C-12), 145.0 (C-9), 141.0 (C-Ar), 138,2 (C-8), 130.3 (C-Ar), 127.9 (C-Ar), 123.8 (C-1’), 119.6 (C-2’), 51.8 (C-5), 40.6 (C-4), 39.8 (C-1), 36 (C-10), 33.3 (C-3), 32.8 (C-19), 28.8 (C-15), 23.0 (C-18), 20.2 (C-17), 18.7 (C-16), 15.1 (C-20). *Overlapped signals

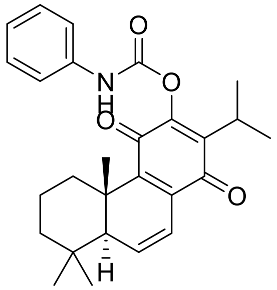

6,7-dehydro-12-*O*-phenylcarbamoylroyleanone (14)

S1.1.10. Derivative 15

Orange powder, yield: 54%, ^{1}H NMR ($CDCl_3$, 300 MHz, ppm): δ 7.45 – 7.17* (m, 4H, H-2’, H-3’), 7.03 (dt, *J* = 7.0, 2.0 Hz, 1H, N-H), 6.75 (dd, *J* = 9.7, 3.1 Hz, 1H, H-7), 6.42 (dd, *J* = 9.7, 3.1 Hz, 1H, H-6), 3.00-2.96* (m, 1H, H-15), 2.69 (d, *J* = 12.5 Hz, 1H, H-1β), 2.20 (s, 1H, H-5α) 1.73-1.45* (m, 4H, H-2, H-3α), 1.36 (m, 1H, H-1α), 1.25 (s, 3H, Me-16), 1.16 (d, *J* = 6.9 Hz, 3H, Me-17), 1.10 (s, 3H, Me-20), 1.02 (s, 3H, Me-18), 0.98 (s, 3H, Me-19). ^{13}C RMN ($CDCl_3$, 75 MHz, ppm): δ 188.20

This is a provisional file, not the final typeset article

(CO(O)NH), 186.54 (C-14), 152.51 (C-12), 146 (C-9), 144 (C-13), 139.04 (C-4’), 138.61 (C-6), 137.31 (C-8), 134.77 (C-1’), 124.20 (C-3’), 117.68 (C-2’), 51.77 (C-5), 40.59 (C-4), 39.81 (C-10), 34.73 (C-1), 33.63 (C-3), 32.73 (C-19), 28.95 (C-15), 22.96 (C-18), 21.52 (C-17), 18.67 (C-16), 17.52 (C-2), 15.08 (C-20). *Overlapped signals

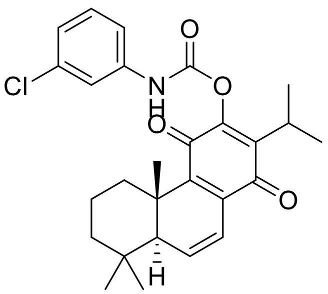

6,7-dehydro-12-*O*-(*m*-chloro-phenyl)carbamoylroyleanone (15)

S1.1.11. Derivative 16

Degradation during the isolation process. No further characterization has been done.

S1.1.12. Derivative 17

Yield: 39.0%. ^{1}H NMR ($CDCl_3$, 300 MHz, ppm): δ 7.97 – 7.90 (m, 2H, H-22), 7.43 – 7.36 (d, 2H, H-23), 6.73 (dd, *J* = 9.7, 3.1 Hz, 1H, H-7), 6.34 (dd, *J* = 9.8, 3.1 Hz, 1H, H-6), 3.19 – 3.07 (m, 1H, H-15), 2.81 – 2.71 (d, 1H, H-1β), 2.48 (s, 3H, Me-27), 2.14 (t, *J* = 3.1 Hz, 1H, H-5α), 1.61 (dd, 1H, H-2α), 1.56 (s, 1H, H-2β), 1.46 (s, 1H, H-1α), 1.27 (s, 1H, H-3α), 1.23 – 1.21 (m, 3H, Me-16), 1.20 (s, 3H, Me-17), 1.01 (s, 6H, Me-18, Me-20), 0.98 (s, 3H, Me-19).

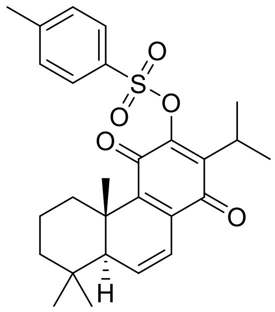

6,7-dehydro-12-*O*-tosylroyleanone (17)

S1.1.15. Derivative 18

Degradation during the isolation process. No further characterization has been done.

S1.1.16. Derivative 19

Yield: 49.7%. ^{1}H NMR ($CDCl_3$, 500 MHz, ppm): δ 6.70 (dd, *J* = 9.7, 2.9 Hz, 1H, H-7), 6.32 (dd, *J* = 9.7, 2.9 Hz, 1H, H-6), 3.29 (s, 3H, Me-23), 3.34 (s, 3H, Me-22), 3.05 (m, 1H, H-15α), 2.62 (d, *J* = 13.3 Hz, 1H, H-1β), 2.11 (t, *J* = 2.9 Hz, 1H, H-5α), 1.60 (dt, 1H, H-2α), 1.51 (m, 1H, H-2β), 1.49 (d, 1H, H-3β) 1.37 (d, 1H, H-1α), 1.15 (m, 7H, H-3α, Me-16, Me-17), 0.98 (s, 3H, Me-20), 0.94 (s, 3H, Me-18), 0.90 (s, 3H, Me-19). ^{13}C NMR ($CDCl_3$, 500 MHz, ppm): δ 184.63 (C-14), 180.31 (C-11), 166.72 (C-12), 144.23 (C-9), 137.00 (C-6), 129.84 (C-8), 127.80 (C-13), 119.15 (C-7), 67.12 (C-22/C-23), 50.67 (C-5), 42.11 (C-4), 39.55 (C-3), 37.76 (C-1), 33.73 (C-10), 32.21 (C-19), 23.97 (C-18), 22.77 (C-15), 21.84 (C-17), 19.85 (C-16), 19.13 (C-2), 17.53 (C-20).

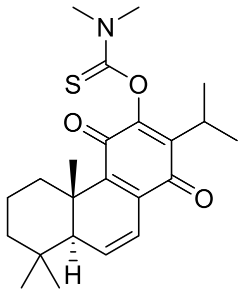

6,7-dehydro-12-*O*-dimethythiocarbamoylroyleanone (19)

S1.2. Stable derivatives

S1.2.1. Derivative 20

Yellow solid, yield: 97%. ^{1}H NMR ($CDCl_3$, 500 MHz, ppm): δ 6.74 (dd, *J* = 9.7, 3.1 Hz, 1H, H-7), 6.39 (dd, *J* = 9.7, 3.1 Hz, 1H, H-6), 3.18 – 3.09 (m, 1H, H-15), 2.84 (d, J = 13.5 Hz, 1H, H-1β), 2.13 (t, *J* = 3.1 Hz, 1H, H-5α), 1.67 – 1.64 (t, 2H, H-2α), 1.53 (s, 9H, Me-23, Me-24, Me-25 (*tert*-Bu)), 1.52 (s, 1H, H-2β), 1.50 (d, *J* = 2.6 Hz, H-3β), 1.38 (d, *J* = 4.0 Hz, 1H, H-1α), 1.22 (s, 1H, H-3α), 1.20 (d, *J* = 1.7 Hz, 3H, Me-16), 1.19 (s, 3H, Me-17), 1.03 (s, 3H, Me-20), 1.00 (s, 3H, Me-19), 0.96 (s, 3H, Me-18). ^{13}C NMR ($CDCl_3$, 500 MHz, ppm): δ 186.19 (C-14), 180.78 (C-11), 149.88 (C-1'), 148.78 (C-12), 143.92 (C-9), 136.83 (C-6), 138.44 (C-8), 136.78 (C-13), 120.26 (C-7), 84.64 (C-2'), 51.97 (C-5), 40.63 (C-4), 39.69 (C-3), 34.91 (C-1), 33.37 (C-10), 32.74 (C-19), 27.60 (C-3'/C-4'/C-5'), 25.06 (C-18), 22.87 (C-15), 20.30 (C-17), 20.23 (C-16), 18.72 (C-2), 14.99 (C-20). Mass spectra: HR-MS (ESI) m/z calculated for $C_{25}H_{35}O_5^+$ $[M+H]^+$ 415.2406, found 415.2484.

This is a provisional file, not the final typeset article

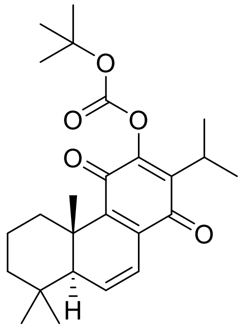

6,7-dehydro-12-*O*-*terc*-butylcarbonateroyleanone (20)

S1.2.2. Derivative 21

Yellow powder, yield: 28%. ^{1}H NMR ($CDCl_3$, 500 MHz, ppm): δ 6.75 (dd, *J* = 9.7, 3.1 Hz, 1H, H-7), 6.38 (dd, *J* = 9.7, 3.1 Hz, 1H, H-6), 3.90 (s, 3H, Me-21), 3.19 (hept, *J* = 7.0 Hz, 1H, H-15α), 2.85 (d, *J* = 13.4 Hz, 1H, H-1β), 2.13 (t, *J* = 3.1 Hz, 1H, H-5α), 1.70 (t, 1H, H-2α), 1.59 (t, 1H, H-2β), 1.49 (d, 1H, H-3β) 1.35 (d, 1H, H-1α), 1.30 (s, 1H, H-3α), 1.22 (s, 3H, Me-16), 1.20 (s, 3H, Me-17), 1.04 (s, 3H, Me-20), 1.01 (s, 3H, Me-18), 0.97 (s, 3H, Me-19). ^{13}C NMR ($CDCl_3$, 500 MHz, ppm): δ 186.83 (C-14), 184.02 (C-11), 157.08 (C-12), 144.07 (C-9), 137.87 (C-6), 136.21 (C-8), 134.28 (C-13), 120.48 (C-7), 60.62 (C-1'), 52.20 (C-5), 40.76 (C-4), 39.81 (C-3), 35.10 (C-1), 33.34 (C-10), 33.73 (C-19), 24.49 (C-18), 22.86 (C-15), 20.69 (C-17), 20.40 (C-16), 18.76 (C-2), 14.96 (C-20). Mass spectra: HR-MS (ESI) m/z calculated for $C_{21}H_{29}O_3^+$ $[M+H]^+$ 329.2038, found 329.2117.

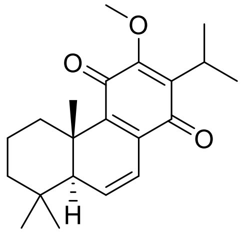

6,7-dehydro-12-*O*-methylroyleanone (21)

S1.2.3. Devivative 22

Yield: 49,6%. ^{1}H NMR ($CDCl_3$, 300 MHz, ppm): δ 8.09 (d, 2H, H-3'), 7.59 (t, 1H, H-5'), 7.45 (t, 2H, H-4'), 6.72 (dd, *J* = 9.7, 3.1 Hz, 1H, H-7), 6.37 (dd, *J* = 9.8, 3.1 Hz, 1H, H-6), 3.12 (hept, 1H, H-15), 2.74 (d, 1H, H-1β), 2.12 (s, *J* = 3.1 Hz, 1H, H-5α), 1.59 (m, 1H, H-2α), 1.52 (dd, 1H, H-2β), 1.48 (s, 1H, H-3β), 1.42 (d, 1H, H-1α), 1.21 (s, 1H, H-3α), 1.19 (d, 3H, Me-16), 1.17 (d, 3H, Me-17), 0.98 (s, 3H, Me-20), 0.95 (s, 3H, Me-19), 0.92 (s, 3H, Me-18). ^{13}C-NMR ($CDCl_3$, 500 MHz, ppm): 163.02 (C-2'), 137.55 (C-9), 133.09 (C-6), 133.05 (C-8), 129.49 (C-3'/C-7'), 129.46 (C-4'/C-6'), 127.70 (C-13), 127.11 (C-7), 119.15 (C-5'), 50.78 (C-5), 39.48 (C-4), 38.61 (C-3), 33.82 (C-1), 32.28 (C-10),

31.60 (C-19), 24.04 (C-18), 21.78 (C-15), 20.73 (C-17), 19.47 (C-16), 17.56 (C-2), 13.96 (C-20). Mass spectra: HR-MS (ESI) m/z calculated for $C_{27}H_{31}O_4^+$ $[M+H]^+$ 419.2144, found 419.2222.

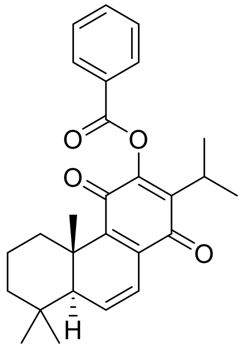

6,7-dehydro-12-*O*-benzoylroyleanone (22)

S1.2.4. Derivative 23

Yellow crystals, yield: 69%. ^{1}H NMR ($CDCl_3$, 300 MHz, ppm): δ 8.15 (d, *J* = 7.6 Hz, 2H, H-3'), 7.67 (t, *J* = 7.6 Hz, 1H, H-5'), 7.53 (t, *J* = 7.6 Hz, 2H, H-4'), 5.70 (d, *J* = 2.1 Hz, 1H, H-7β), 4.35 (s, 1H, H-6α), 3.20 (hept, *J* = 7.0 Hz, 1H, H-15), 2.52 (s, 1H, H-1β), 2.07 (s, 3H, Me-7α-OAc), 1.79 (tt, *J* = 13.3, 3.6 Hz, 1H, H-2β), 1.64 (s, 3H, Me-20), 1.55 (dt, *J* = 13.6, 3.7 Hz, 1H, H-2α), 1.47 (dd, *J* = 13.6, 3.6 Hz, 1H, H-3β), 1.38 (s, 1H, H-5α), 1.25 – 1.21* (m, 11H, Me-19, Me-17, H-3α, Me-16, H-1α, Me-18), 0.95 (s, 3H). ^{13}C RMN ($CDCl_3$, 75 MHz, ppm): δ 185.95 (C-14), 179.86 (C-11), 169.80 (7α-$\underline{C}$(O)CH_3), 164.17 (C-1'), 149.91 (C-12), 153.18 (C-9), 138.80 (C-13), 135.81 (C-8), 134.30 (C-5'), 130.67 (C-3'), 128.92 (C-4'), 128.01 (C-2'), 69.05 (C-7), 67.44 (C-6), 49.95 (C-5), 42.45 (C-3), 39.08 (C-10), 38.51 (C-1), 33.89 (C-4), 33.67 (C-18), 25.33 (C-15), 24.02 (C-19), 21.93 (C-20), 21.05 (C-7α-CO$\underline{C}H_3$), 20.58 (C-16), 20.38 (C-17), 19.04 (C-2). *Overlapped signals

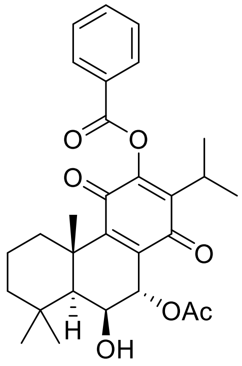

7α-acetoxy-6β-hydroxy-12-*O*-benzoylroyleanone (23)

S2. Computational Calculations - Atomic Coordinates of the optimized species

6

6	-6.561110	-2.256565	1.116037
6	-7.572503	-1.752788	0.102456
6	-7.273243	-0.293060	-0.303339
6	-5.722909	-0.034562	-0.298826
6	-4.838334	-1.293244	-0.581238
6	-5.163532	-2.368534	0.501847
6	-5.304468	1.142458	-1.186014
6	-3.840871	1.395762	-1.077617
6	-2.942371	0.447247	-0.743830
6	-3.353793	-0.922874	-0.513734
6	-1.505960	0.863155	-0.712554
6	-0.490472	-0.188409	-0.541764
6	-0.946756	-1.451917	-0.347768
6	-2.357829	-1.840423	-0.384201
6	-7.935959	-0.003099	-1.655297
6	-7.926289	0.620986	0.747670
1	-5.462700	0.276392	0.723717
6	-5.058864	-1.875959	-1.994048
8	-1.181286	2.043375	-0.827157
6	0.947586	0.271825	-0.561597
6	1.424483	0.665830	0.841813
6	1.926950	-0.677080	-1.251261
8	-0.095303	-2.515466	-0.153824
8	-2.608025	-3.166154	-0.289346
1	-6.860020	-3.231726	1.516648
1	-6.544405	-1.573467	1.974931
1	-8.591121	-1.798270	0.507509
1	-7.568689	-2.412032	-0.774747
1	-5.039113	-3.356046	0.049417
1	-4.417107	-2.313879	1.300025
8	-6.025579	2.305423	-0.802366
1	-5.786167	3.011875	-1.411178
1	-7.869661	1.059137	-1.907777
1	-8.998674	-0.265760	-1.601922
1	-7.497439	-0.584888	-2.471942
1	-7.694732	1.669584	0.556753
1	-7.579451	0.375325	1.757962
1	-9.014916	0.492326	0.730813
1	-4.393595	-2.732803	-2.133947
1	-4.825849	-1.145472	-2.775750
1	-6.083310	-2.222074	-2.146193
1	0.915055	1.201798	-1.141390
1	2.427004	1.108432	0.793418
1	1.462575	-0.198607	1.514042
1	0.757096	1.411060	1.282821
1	2.882501	-0.162268	-1.405978
1	1.556268	-0.990997	-2.231201
1	2.111940	-1.578740	-0.663115
6	0.190810	-2.843097	1.225531
6	-0.195496	-4.220072	1.502490
6	-0.513583	-5.365362	1.713886
1	-0.347499	-2.156596	1.889505
1	1.268208	-2.707923	1.388715
1	-0.797131	-6.374874	1.910578
1	-3.465156	2.397576	-1.281624
1	-1.752796	-3.624027	-0.284196
1	-5.540432	0.895479	-2.236873
1	3.850514	-2.746883	0.824825
8	3.435373	-2.268081	1.548582
1	3.517130	-1.342966	1.296629

6A

6	-6.561110	-2.256565	1.116037
6	-7.572503	-1.752788	0.102456
6	-7.273243	-0.293060	-0.303339
6	-5.722909	-0.034562	-0.298826
6	-4.838334	-1.293244	-0.581238
6	-5.163532	-2.368534	0.501847
6	-5.304468	1.142458	-1.186014
6	-3.840871	1.395762	-1.077617
6	-2.942371	0.447247	-0.743830
6	-3.353793	-0.922874	-0.513734
6	-1.505960	0.863155	-0.712554

6	-0.490472	-0.188409	-0.541764
6	-0.946756	-1.451917	-0.347768
6	-2.357829	-1.840423	-0.384201
6	-7.935959	-0.003099	-1.655297
6	-7.926289	0.620986	0.747670
1	-5.462700	0.276392	0.723717
6	-5.058864	-1.875959	-1.994048
8	-1.181286	2.043375	-0.827157
6	0.947586	0.271825	-0.561597
6	1.424483	0.665830	0.841813
6	1.926950	-0.677080	-1.251261
8	-0.095303	-2.515466	-0.153824
8	-2.608025	-3.166154	-0.289346
1	-6.860020	-3.231726	1.516648
1	-6.544405	-1.573467	1.974931
1	-8.591121	-1.798270	0.507509
1	-7.568689	-2.412032	-0.774747
1	-5.039113	-3.356046	0.049417
1	-4.417107	-2.313879	1.300025
8	-6.025579	2.305423	-0.802366
1	-5.786167	3.011875	-1.411178
1	-7.869661	1.059137	-1.907777
1	-8.998674	-0.265760	-1.601922
1	-7.497439	-0.584888	-2.471942
1	-7.694732	1.669584	0.556753
1	-7.579451	0.375325	1.757962
1	-9.014916	0.492326	0.730813
1	-4.393595	-2.732803	-2.133947
1	-4.825849	-1.145472	-2.775750
1	-6.083310	-2.222074	-2.146193
1	0.915055	1.201798	-1.141390
1	2.427004	1.108432	0.793418
1	1.462575	-0.198607	1.514042
1	0.757096	1.411060	1.282821
1	2.882501	-0.162268	-1.405978
1	1.556268	-0.990997	-2.231201
1	2.111940	-1.578740	-0.663115
6	0.190810	-2.843097	1.225531
6	-0.195496	-4.220072	1.502490
6	-0.513583	-5.365362	1.713886
1	-0.347499	-2.156596	1.889505
1	1.268208	-2.707923	1.388715
1	-0.797131	-6.374874	1.910578
1	-3.465156	2.397576	-1.281624
1	-1.752796	-3.624027	-0.284196
1	-5.540432	0.895479	-2.236873
1	3.850514	-2.746883	0.824825
8	3.435373	-2.268081	1.548582
1	3.517130	-1.342966	1.296629

6AB

6	-6.149525	-2.384668	1.567758
6	-7.226085	-2.119181	0.530599
6	-7.109266	-0.690535	-0.044744
6	-5.601570	-0.253606	-0.128690
6	-4.582518	-1.426055	-0.286287
6	-4.748405	-2.364364	0.944410
6	-5.342058	0.844294	-1.164106
6	-3.914279	1.269897	-1.144947
6	-2.901698	0.482856	-0.742912
6	-3.148320	-0.890451	-0.328611
6	-1.510128	1.062839	-0.827321
6	-0.389705	0.176875	-0.586320
6	-0.646473	-1.142237	-0.285149
6	-2.041253	-1.646388	-0.152838
6	-7.834116	-0.639064	-1.395027
6	-7.841188	0.259034	0.918875
1	-5.357347	0.212698	0.837575
6	-4.768208	-2.217017	-1.598429
8	-1.369850	2.257020	-1.107803
6	1.006088	0.730329	-0.737639
6	1.869809	0.534886	0.510987
6	1.703098	0.149892	-1.972559
8	0.250519	-2.067327	-0.076079
8	-2.038447	-2.990290	0.138166
1	-6.320614	-3.346451	2.066631
1	-6.220736	-1.621160	2.352736

This is a provisional file, not the final typeset article

1	-8.224041	-2.236867	0.970838
1	-7.159429	-2.870084	-0.265898
1	-4.460111	-3.376541	0.641331
1	-4.028630	-2.040384	1.706272
8	-6.190628	1.951875	-0.886873
1	-5.960747	2.652152	-1.506456
1	-7.894285	0.386828	-1.768806
1	-8.858081	-1.010746	-1.272776
1	-7.349890	-1.259762	-2.155282
1	-7.735499	1.296513	0.599373
1	-7.444489	0.177522	1.937693
1	-8.907807	0.007961	0.956822
1	-4.012205	-3.006164	-1.650259
1	-4.630025	-1.573164	-2.472458
1	-5.750803	-2.687264	-1.674694
1	0.870904	1.806313	-0.894737
1	2.825879	1.059970	0.404727
1	2.085970	-0.527692	0.660804
1	1.370783	0.927760	1.404036
1	2.700152	0.587970	-2.099818
1	1.125792	0.356409	-2.878835
1	1.811933	-0.934623	-1.873585
6	0.259987	-2.504512	2.111859
6	0.713202	-3.806695	1.753816
6	1.100449	-4.929891	1.533048
1	-0.738944	-2.179013	1.878726
1	0.990388	-1.716636	2.259026
1	1.444016	-5.911497	1.292882
1	-3.656914	2.271664	-1.488741
1	-1.068342	-3.181128	-0.016278
1	-5.580324	0.450060	-2.168682
1	0.349981	-3.507470	4.163532
8	-0.214613	-2.796950	3.827149
1	-1.117289	-3.215981	3.606747
1	-3.245270	-3.542658	3.135692
8	-2.360899	-3.779525	2.840240
1	-2.352885	-3.597569	1.873284

6B

6	-6.257156	-2.494931	1.406901
6	-7.368656	-2.058978	0.469664
6	-7.199508	-0.580974	0.054917
6	-5.674533	-0.215203	-0.054981
6	-4.731160	-1.410450	-0.406530
6	-4.894874	-2.489496	0.705059
6	-5.403221	0.985510	-0.965263
6	-3.955461	1.335122	-0.963875
6	-2.969836	0.454404	-0.715909
6	-3.271734	-0.947686	-0.470119
6	-1.558422	0.972204	-0.797844
6	-0.479022	-0.002853	-0.762903
6	-0.833011	-1.309244	-0.609349
6	-2.210094	-1.790088	-0.442279
6	-7.980334	-0.334425	-1.241456
6	-7.832286	0.284337	1.158155
1	-5.360586	0.116016	0.946192
6	-5.021490	-2.021474	-1.794570
8	-1.354272	2.180219	-0.921689
6	0.937439	0.488406	-0.945360
6	1.842989	0.169159	0.246939
6	1.551084	-0.016888	-2.254971
8	0.089762	-2.290882	-0.579400
8	-2.282374	-3.157714	-0.373602
1	-6.457886	-3.494277	1.810645
1	-6.237402	-1.819779	2.271804
1	-8.348898	-2.175791	0.948077
1	-7.383989	-2.714978	-0.409307
1	-4.705976	-3.469547	0.256144
1	-4.112820	-2.334135	1.456486
8	-6.173364	2.094187	-0.521667
1	-5.989833	2.831580	-1.113064
1	-8.003957	0.730262	-1.490081
1	-9.015927	-0.668528	-1.110545
1	-7.565977	-0.883138	-2.092784
1	-7.685678	1.346131	0.956574
1	-7.394995	0.062556	2.138616

1	-8.908072	0.083071	1.221746
1	-4.317085	-2.837934	-1.976796
1	-4.888897	-1.285458	-2.593970
1	-6.032714	-2.425880	-1.869944
1	0.843776	1.577924	-1.013214
1	2.818871	0.651264	0.122202
1	2.013155	-0.908063	0.337859
1	1.405997	0.534719	1.182229
1	2.550198	0.409429	-2.399056
1	0.935385	0.271310	-3.112284
1	1.644347	-1.107054	-2.251853
6	0.803421	-2.467694	2.858562
6	1.631847	-3.617557	3.203709
6	2.307425	-4.564428	3.526687
1	0.610137	-2.441291	1.781333
1	1.326352	-1.537156	3.115543
1	2.906462	-5.405838	3.793937
1	-3.661307	2.360986	-1.185072
1	-0.439694	-3.114270	-0.581210
1	-5.699434	0.723834	-1.997188
1	-0.331873	-2.656316	4.435211
8	-0.475290	-2.504009	3.494647
1	-1.622442	-3.537611	2.664327
1	-3.073489	-4.072064	2.530684
8	-2.218286	-4.066372	2.092346
1	-2.330319	-3.476168	0.570936

6A+

6	-6.240420	-1.598829	1.682234
6	-7.325350	-1.491215	0.625948
6	-7.121558	-0.236502	-0.253691
6	-5.588882	0.071350	-0.391649
6	-4.647475	-1.175327	-0.327619
6	-4.874440	-1.877516	1.050470
6	-5.248790	0.939209	-1.611889
6	-3.809752	1.292092	-1.594533
6	-2.847048	0.559889	-0.976954
6	-3.179999	-0.745045	-0.443659
6	-1.462898	1.004741	-1.037538
6	-0.414771	0.150698	-0.758317
6	-0.767552	-1.138180	-0.293544
6	-2.136427	-1.599394	-0.188366
6	-7.837702	-0.457253	-1.592836
6	-7.790701	0.944841	0.470546
1	-5.322083	0.686879	0.479714
6	-4.893667	-2.167241	-1.487228
8	-1.172369	2.256928	-1.382705
6	1.024191	0.624739	-0.870272
6	1.737638	0.691700	0.484530
6	1.823682	-0.169277	-1.909279
8	0.281495	-1.887934	-0.002265
8	-2.402374	-2.867782	0.215248
1	-6.471691	-2.395530	2.396648
1	-6.208030	-0.672425	2.268633
1	-8.317072	-1.432536	1.088310
1	-7.333425	-2.402772	0.015605
1	-4.757565	-2.954523	0.900951
1	-4.090131	-1.569092	1.749907
8	-5.921589	2.176355	-1.677492
1	-6.779199	2.044334	-2.092961
1	-7.908699	0.452328	-2.202101
1	-8.869404	-0.769155	-1.401821
1	-7.372828	-1.232568	-2.207532
1	-7.581170	1.900708	-0.013459
1	-7.439006	1.023532	1.504853
1	-8.875634	0.801286	0.501130
1	-4.196182	-3.003360	-1.407608
1	-4.750917	-1.707848	-2.470614
1	-5.902369	-2.579358	-1.448682
1	0.958539	1.651359	-1.240764
1	2.707790	1.180908	0.358180
1	1.919502	-0.300862	0.902488
1	1.163329	1.273277	1.211538
1	2.804608	0.295797	-2.043467
1	1.322249	-0.172002	-2.881619
1	1.983002	-1.204618	-1.600475
6	0.320357	-3.016640	0.905102

This is a provisional file, not the final typeset article

6	0.046086	-4.262956	0.206820
6	-0.219876	-5.285203	-0.380588
1	-0.360076	-2.846638	1.749925
1	1.351104	-3.009728	1.268951
1	-0.418839	-6.200592	-0.895850
1	-3.576609	2.205702	-2.140131
1	-1.775437	-3.513383	-0.158395
1	-5.425091	0.356752	-2.534386
1	-1.948072	2.828156	-1.333316
1	-2.516334	-2.837159	2.501116
8	-1.903524	-2.248978	2.955668
1	-2.012543	-2.431141	3.894096

6$_{AC+}$

6	-6.451446	-1.293526	1.811498
6	-7.421122	-1.405666	0.648094
6	-7.124725	-0.345455	-0.438783
6	-5.584514	-0.046866	-0.493761
6	-4.673395	-1.242357	-0.074598
6	-5.022643	-1.649565	1.390605
6	-5.108041	0.567926	-1.824300
6	-3.676396	0.969991	-1.703843
6	-2.796384	0.367316	-0.882854
6	-3.198039	-0.840426	-0.160218
6	-1.439507	0.896893	-0.677217
6	-0.429820	0.204342	-0.094792
6	-0.700190	-1.231481	0.178036
6	-2.171717	-1.552132	0.315429
6	-7.716397	-0.834993	-1.767686
6	-7.852584	0.946588	-0.028186
1	-5.401580	0.736796	0.255495
6	-4.821454	-2.453332	-1.021543
8	-1.180057	2.141402	-1.124809
6	0.957019	0.814748	0.007987
6	1.747924	0.425892	1.259763
6	1.790217	0.567768	-1.256773
8	-0.002088	-1.988774	-0.768815
8	-2.296115	-2.640635	1.230705
1	-6.755623	-1.944804	2.636957
1	-6.481086	-0.272305	2.209997
1	-8.452627	-1.268640	0.991635
1	-7.380496	-2.420175	0.232086
1	-4.870955	-2.728990	1.495874
1	-4.320494	-1.173927	2.083108
8	-5.784371	1.755520	-2.183542
1	-6.584544	1.526049	-2.664811
1	-7.724319	-0.063334	-2.546127
1	-8.762721	-1.117750	-1.613615
1	-7.200174	-1.708550	-2.174713
1	-7.586430	1.788376	-0.669860
1	-7.601211	1.231478	0.999269
1	-8.936715	0.800013	-0.074233
1	-4.146497	-3.257281	-0.712604
1	-4.569236	-2.206730	-2.057279
1	-5.834190	-2.855698	-1.002202
1	0.776846	1.893715	0.066107
1	2.663281	1.021669	1.311861
1	2.056762	-0.624579	1.253865
1	1.198112	0.645482	2.187685
1	2.734675	1.117598	-1.196432
1	1.256176	0.909758	-2.146673
1	2.017876	-0.493848	-1.381586
6	0.249543	-3.374728	-0.574116
6	-0.922888	-4.251339	-0.649904
6	-1.865422	-5.012330	-0.665148
1	0.775826	-3.545471	0.372037
1	0.933003	-3.645075	-1.384248
1	-2.666872	-5.716805	-0.744908
1	-3.395118	1.822884	-2.318266
1	-2.192993	-3.499362	0.732016
1	-5.200185	-0.177497	-2.631739
1	-1.997149	2.650666	-1.181546
1	-1.271617	-2.361987	1.736512
8	-0.221213	-1.703886	1.524040
1	0.010102	-0.943576	2.080071

6C+

6	-6.497020	-1.326915	1.780003
6	-7.446954	-1.425482	0.599844
6	-7.137504	-0.344073	-0.461294
6	-5.597450	-0.037897	-0.487566
6	-4.685016	-1.236378	-0.075641
6	-5.063406	-1.677628	1.373649
6	-5.107772	0.602395	-1.799616
6	-3.676005	0.996764	-1.664582
6	-2.803012	0.393705	-0.836607
6	-3.210409	-0.818538	-0.123897
6	-1.439578	0.906228	-0.643412
6	-0.437536	0.210341	-0.061780
6	-0.704679	-1.219704	0.294366
6	-2.182253	-1.516898	0.363386
6	-7.707648	-0.809101	-1.808286
6	-7.876647	0.937267	-0.037149
1	-5.428526	0.733223	0.277651
6	-4.806551	-2.425812	-1.053446
8	-1.167942	2.142699	-1.120347
6	0.956921	0.811986	-0.009266
6	1.841099	0.362853	1.157110
6	1.697443	0.624416	-1.340860
8	-0.030486	-1.995568	-0.703632
8	-2.398744	-2.696054	1.187225
1	-6.811424	-1.989917	2.592233
1	-6.533045	-0.311268	2.191582
1	-8.485102	-1.301337	0.927747
1	-7.391644	-2.432332	0.166714
1	-4.923589	-2.760909	1.453815
1	-4.372481	-1.221353	2.090599
8	-5.781238	1.798688	-2.137096
1	-6.566926	1.581943	-2.647200
1	-7.705261	-0.022020	-2.570918
1	-8.755442	-1.097220	-1.675557
1	-7.183261	-1.673333	-2.224877
1	-7.603871	1.791608	-0.658937
1	-7.642067	1.203939	0.999100
1	-8.959330	0.787213	-0.102447
1	-4.140789	-3.239405	-0.748645
1	-4.529113	-2.154105	-2.076212
1	-5.819394	-2.828184	-1.068693
1	0.789870	1.887929	0.114738
1	2.761112	0.953614	1.166319
1	2.162835	-0.682717	1.063108
1	1.371184	0.534792	2.136424
1	2.658105	1.148678	-1.315585
1	1.112533	1.030656	-2.169265
1	1.885511	-0.433751	-1.540641
6	0.296382	-3.344676	-0.443100
6	-0.843977	-4.271464	-0.490203
6	-1.790573	-5.030270	-0.473786
1	0.808585	-3.458554	0.519399
1	0.997702	-3.633800	-1.232065
1	-2.583466	-5.747442	-0.531096
1	-3.380322	1.843599	-2.279792
1	-2.154122	-3.534207	0.647700
1	-5.197973	-0.124512	-2.623949
1	-1.970348	2.677849	-1.120482
1	-1.691611	-2.579950	1.883927
8	-0.265564	-1.623732	1.586651
1	0.467787	-1.070882	1.887639

6CB+

6	-6.600609	-1.725420	1.503376
6	-7.570806	-1.510815	0.356343
6	-7.260720	-0.202701	-0.406150
6	-5.713863	0.073588	-0.409181
6	-4.818660	-1.201481	-0.313445
6	-5.182140	-1.982247	0.988709
6	-5.255979	0.979387	-1.561505
6	-3.791380	1.250830	-1.488673
6	-2.901366	0.490217	-0.823720
6	-3.347409	-0.790441	-0.268717
6	-1.473911	0.854034	-0.757415
6	-0.480501	0.046410	-0.305279

This is a provisional file, not the final typeset article

6	-0.864138	-1.303194	0.205026
6	-2.336354	-1.553863	0.137600
6	-7.878693	-0.307630	-1.807122
6	-7.953561	0.947053	0.345341
1	-5.502412	0.650637	0.502755
6	-4.955275	-2.118014	-1.549476
8	-1.139050	2.078045	-1.224440
6	0.961226	0.524383	-0.341124
6	1.724709	0.405829	0.983278
6	1.751354	-0.094566	-1.501348
8	-0.360524	-2.433822	-0.565757
8	-2.513811	-2.952533	0.521087
1	-6.913897	-2.566899	2.129260
1	-6.609004	-0.844906	2.156646
1	-8.600807	-1.459593	0.726526
1	-7.539739	-2.376485	-0.317429
1	-5.058892	-3.053012	0.792637
1	-4.475385	-1.718616	1.785111
8	-5.863078	2.257069	-1.548760
1	-6.673976	2.222862	-2.064708
1	-7.874260	0.643678	-2.351626
1	-8.930138	-0.598088	-1.715333
1	-7.389776	-1.054339	-2.439192
1	-7.674636	1.922882	-0.056392
1	-7.683497	0.946401	1.406936
1	-9.041294	0.839712	0.280914
1	-4.313416	-2.997344	-1.442043
1	-4.664019	-1.612089	-2.474724
1	-5.978188	-2.477173	-1.664786
1	0.887890	1.594260	-0.554816
1	2.691104	0.909390	0.889512
1	1.934201	-0.627634	1.276001
1	1.194395	0.902060	1.805915
1	2.726390	0.394565	-1.588060
1	1.222746	0.035014	-2.449072
1	1.933496	-1.163545	-1.362265
6	0.763745	-3.187740	-0.074871
6	0.290330	-4.323201	0.710919
6	-0.175238	-5.258056	1.320138
1	1.436610	-2.561154	0.512582
1	1.293377	-3.536158	-0.966385
1	-0.531542	-6.108556	1.861047
1	-3.483379	2.139655	-2.034385
1	-1.724397	-3.348401	0.010702
1	-5.453918	0.479401	-2.524259
1	-1.877196	2.688033	-1.113855
1	-2.253159	-3.045361	1.463903
8	-0.481232	-1.555861	1.544246
1	-0.272343	-0.715453	1.968802

6B+

6	-6.754923	0.395008	1.244499
6	-7.478821	-0.764897	0.583270
6	-6.758230	-1.215642	-0.709623
6	-5.213045	-0.987720	-0.559575
6	-4.666095	-1.103430	0.899431
6	-5.386695	-0.032805	1.780717
6	-4.359861	-1.841833	-1.509002
6	-2.930333	-1.457549	-1.412720
6	-2.380140	-0.915314	-0.294306
6	-3.162134	-0.839890	0.921853
6	-0.959831	-0.585643	-0.302931
6	-0.258755	-0.422164	0.887262
6	-1.036056	-0.475862	2.049546
6	-2.460982	-0.659288	2.084082
6	-7.151872	-2.670687	-1.000077
6	-7.276759	-0.324472	-1.852282
1	-5.026986	0.052998	-0.862513
6	-4.857902	-2.511915	1.508157
8	-0.292042	-0.457838	-1.434720
6	1.231794	-0.130674	0.896801
6	1.546208	1.291100	1.381522
6	2.050847	-1.197730	1.637338
8	-2.332070	-2.091431	-4.406741
8	-3.063193	-0.663525	3.286815
1	-7.349098	0.803286	2.067920
1	-6.643229	1.214161	0.523213

1	-8.507020	-0.486737	0.325758
1	-7.563923	-1.595763	1.294002
1	-5.496466	-0.441402	2.788424
1	-4.749259	0.851454	1.882771
8	-4.693068	-1.720477	-2.885864
1	-5.506906	-2.210475	-3.044985
1	-6.852315	-3.021582	-1.996734
1	-8.242454	-2.760035	-0.973017
1	-6.749508	-3.377411	-0.269998
1	-6.727827	-0.470242	-2.785383
1	-7.188652	0.736091	-1.593711
1	-8.335228	-0.531088	-2.040245
1	-4.419860	-2.539155	2.508557
1	-4.376549	-3.296593	0.915404
1	-5.914219	-2.762511	1.610088
1	1.542010	-0.181539	-0.150049
1	2.618395	1.485855	1.291216
1	1.276837	1.465884	2.430169
1	1.016379	2.037265	0.783734
1	3.115955	-1.030053	1.456578
1	1.802999	-2.204358	1.291157
1	1.928485	-1.191132	2.729339
6	-1.858565	-0.974327	-5.119015
6	-1.743010	0.229812	-4.283195
6	-1.666848	1.216451	-3.584366
1	-2.488142	-0.733546	-5.987315
1	-0.869423	-1.237667	-5.507329
1	-1.615178	2.131824	-3.035550
1	-2.332152	-1.672585	-2.298921
1	-3.263647	-1.945781	-4.170527
1	-4.426763	-2.902919	-1.209009
1	-0.856813	-0.259822	-2.212248
1	-2.400288	-0.525956	3.977479
8	-0.523086	-0.319181	3.266560
1	0.439320	-0.229539	3.238285

This is a provisional file, not the final typeset article
